# Hypoglycemic Activity of Aqueous Extract of Latex from *Hancornia speciosa* Gomes: A Study in Zebrafish and In Silico

**DOI:** 10.3390/ph14090856

**Published:** 2021-08-26

**Authors:** Rosana Tomazi, Ângela Costa Figueira, Adriana Maciel Ferreira, Diego Quaresma Ferreira, Gisele Custódio de Souza, Wandson Braamcamp de Souza Pinheiro, José Rodrigues Pinheiro Neto, Geilson Alcantara da Silva, Henrique Barros de Lima, Lorane Izabel da Silva Hage-Melim, Arlindo César Matias Pereira, José Carlos Tavares Carvalho, Sheylla Susan Moreira da Silva de Almeida

**Affiliations:** 1 Programa de Pós-Graduação em Biodiversidade e Biotecnologia da Rede Bionorte (Ppg-Bionorte), Instituto Federal de Educação, Ciência e Tecnologia do Amapá (IFAP), Rodovia BR-210, km 03, S/n—Brasil Novo, Macapá 68909-398, AP, Brazil; rosana.tomazi@ifap.edu.br (R.T.); cost_angela@hotmail.com (Â.C.F.); 2 Laboratório de Pesquisa em Fármacos, Departamento de Ciências Biológicas e da Saúde, Universidade Federal do Amapá (UNIFAP), Rod. Juscelino Kubitschek, km 02—Jardim Marco Zero, Macapá 68903-419, AP, Brazil; adrianmaciel@gmail.com (A.M.F.); diegomendesmauer@hotmail.com (D.Q.F.); gi.custodio.souza@gmail.com (G.C.d.S.); arlindo.matias@usp.br (A.C.M.P.); 3 Laboratório de Farmacognosia e Fitoquímica, Departamento de Ciências Biológicas e da Saúde, Universidade Federal do Amapá (UNIFAP), Rod. Juscelino Kubitschek, km 02—Jardim Marco Zero, Macapá 68903-419, AP, Brazil; sheyllasusan@yahoo.com.br; 4 Laboratório de Química Industrial, Instituto de Química, Universidade Federal do Pará (UFPA), Rua. Augusto Corrêa, Guamá, 01, Belém 66075-110, AP, Brazil; wbraamcamp@hotmail.com (W.B.d.S.P.); pinheiro.jose.neto@gmail.com (J.R.P.N.); geilsonalcantara@gmail.com (G.A.d.S.); 5 Laboratório de Química Medicinal, Departamento de Ciências Biológicas e da Saúde, Universidade Federal do Amapá (UNIFAP), Rod. Juscelino Kubitschek, km 02—Jardim Marco Zero, Macapá 68903-419, AP, Brazil; hbarros07.hb@gmail.com (H.B.d.L.); lorane@unifap.br (L.I.d.S.H.-M.)

**Keywords:** *Hancornia speciosa* Gomes, 1-*O*-methyl-myoinositol, diabetes, zebrafish

## Abstract

*Hancornia speciosa* Gomes is a tree native to Brazil and has therapeutic potential for several diseases. Ethnopharmacological surveys have reported that the plant is used as a hypoglycemic agent and to lose weight. This study aimed to evaluate the effects of the aqueous extract from H. speciosa latex (LxHs) in a zebrafish model of diabetes. The extract was evaluated through high-performance thin-layer chromatography (HTPLC), nuclear magnetic resonance (NMR), and Fourier-transform infrared spectroscopy (FT-IR). We then tested treatments with LxHs (500, 1000, and 1500 mg/kg) by assessing blood glucose levels in alloxan-induced diabetic animals, and metformin was used as a control. The toxicity was evaluated through histopathology of the pancreas and biochemical assessment of serum levels of AST, ALT, creatinine, and urea. The extract was also assessed for acute toxicity through several parameters in embryos and adult animals. Finally, we performed in silico analysis through the SEA server and docking using the software GOLD. The phytochemical study showed the compounds cornoside, dihydrocornoide, and 1-O-methyl-myoinositol (bornesitol). The treatment with all doses of LxHs significantly decreased alloxan-induced hyperglycemia without any significant histological or biochemical abnormalities. No significant frequency of teratogenesis was observed in the embryos exposed to the extract, and no significant behavioral changes or deaths were observed in adult animals. In silico, the results showed a potential interaction between inositol and enzymes involved in carbohydrates’ metabolism. Overall, the results show a hypoglycemic activity of the extract in vivo, with no apparent toxicity. The computational studies suggest this could be at least partially due to the presence of bornesitol, since inositols can interact with carbohydrates’ enzymes.

## 1. Introduction

Plants are used for therapeutic purposes in the healing or treatment of a range of diseases. However, a significant part of traditional uses is not supported by scientific studies [1]. In Brazil, the use of plants is widespread in folk medicine inside traditional communities. One of these plants is *Hancornia speciosa* Gomes, popularly known as “mangabeira”. Native to this country, this species is found throughout the Amazon forest, the Brazilian semiarid region (called “caatinga”), and the Atlantic forest [2]. Despite its ecological, traditional, and research importance, this species is currently endangered [3].

Phytochemical studies have been performed with different tree parts, including leaves, barks, fruit, and latex. The leaves of H. speciosa Gomes are reported to have terpenoids, steroids, tannins [4,5], and xanthines [5]. In the fruits are found phenols (flavonoids, condensed tannins) and alkaloids [6,7]. Moraes and coworkers reported flavonoids and tannins (proanthocyanidins) in the barks’ ethanol extract [8]. In 2016, Neves and coworkers reported the occurrence of phenols in the tree’s latex [9].

In literature, reports indicate angiogenic and osteogenic potential in the latex, without cytotoxicity or genotoxicity [9,10,11,12]. Although this latex is similar to the seringueira’s latex (*Hevea brasiliensis* L.), the former is most often used against tuberculosis, ulcer, fungi infection, and some inflammatory conditions [13]. The latex of mangabeira is often consumed mixed with water to treat inflammatory diseases and other conditions; despite the latex’s frequent usage, studies in the literature about its potential are still lacking compared to the other three parts [14]. The drug discovery process also relies on the safety of compounds assessed, and zebrafish have been extensively used for this purpose [15,16,17].

Diabetes mellitus (DM) is a syndrome resulting from dysfunctional metabolism of carbohydrates, fats, and proteins that will cause hyperglycemia—its main feature [18]. The global increase in its prevalence makes it a public health burden worldwide [19]. According to Miranda [20], the pathophysiology of diabetes is triggered by both genetic and environmental factors. Current diabetes treatments meet some of the patients’ needs. Still, new antidiabetic drugs are in growing demand. Worldwide, the use of plant-derived compounds has been of pivotal importance in the process of drug discovery in the search for new treatments [21]. Some ethnopharmacology surveys report that *H. speciosa* Gomes is used in folk medicine to treat diabetes and lose weight [22,23]. Preclinical studies have validated its hypoglycemic activity in vitro [24] and in vivo [25]; however, both studies have used extracts from the leaves, and currently, there is no study assessing the extract from the latex, which is also used in folk medicine as reported by [13].

The increased research on natural products and their derivatives has shown promising antidiabetic compounds for developing potential new therapies [26]. An example of such compounds is the inositols, molecules that have been gaining attention for the treatment of diabetes; they are saturated cyclic polyols with six hydroxylated carbons (hexahydroxy-cyclohexane) with nine stereoisomers. Inositols are found in several plants and foods—such as beans and fruits—as inositol derivatives, such as hexaphosphates (phytic acid) or their salt derivatives (phytates) [27]. Myoinositol is the most commonly found in biological systems; it is highly water-soluble and is involved in crucial physiological processes, including insulin release [28]. 1-O-Methyl-myoinositol (bornesitol) is a myoinositol derivative found in plants, including *Hancornia speciosa* Gomes, that has a potential use against chronic diseases such as diabetes [29,30].

Zebrafish have been shown to be a powerful tool for drug discovery [31,32,33,34,35]. Among their advantages are the low amount of drugs used, low maintenance costs, shorter test periods, easy control of experimental conditions, and the conserved genetic similarity between zebrafish and humans (approximately 70%) [15,36]. The zebrafish pancreas has the same functions as the mammal pancreas concerning glucose homeostasis, including the production and secretion of insulin, glucagon, somatostatin, and digestive enzymes such as amylase [37].

In this sense, using alloxan-induced diabetes in zebrafish, this study aimed to evaluate the chemical composition, hypoglycemic effect, and toxicity of the aqueous extract from the latex of *H. speciosa*.

## 2. Results

### 2.1. Phytochemical Analysis

**Figure 1:**
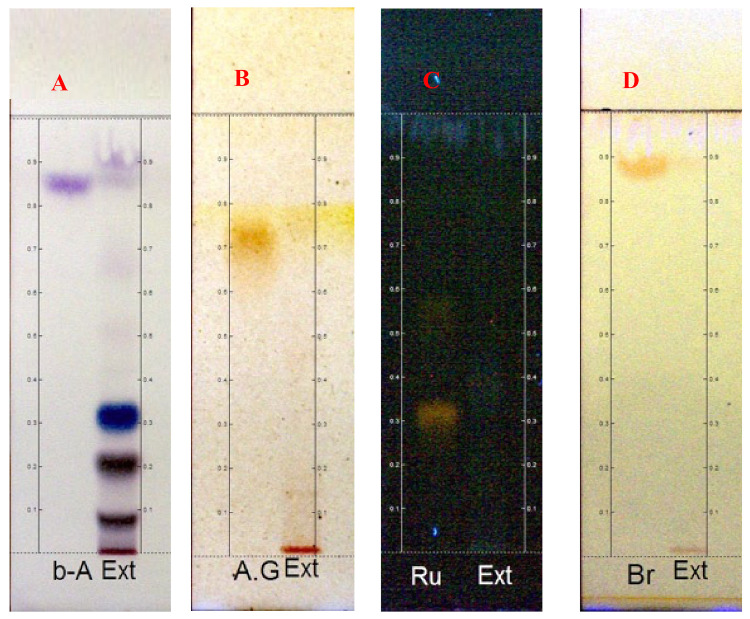
Comparative HPTLC plates of standard solutions and the aqueous from *Hancornia speciosa* Gomes (LxHs). (**A**): shows a plate comparing the extract (Ext) with a standard β-amyrin (b-A) solution, viewed under white light. (**B**): shows a plate comparing Ext with a standard gallic acid (A.G) solution, viewed under UV 254 nm radiation. (**C**): shows a plate comparing Ext with standard rutin (Ru) solution, viewed under UV 366 nm radiation. (**D**): shows a plate comparing Ext with a standard brucin (Br) solution, viewed under UV 254 nm radiation.

HPLC and derivatization were performed to detect groups of compounds in the latex’s aqueous extract. After derivatization with VAS, the occurrence of blue/purple bands, characteristic of terpenes or steroids, was observed; the same was observed in the control plate with β-amyrin (Figure 1A). The occurrence of bands tending to red could suggest the presence of phenolic compounds.

After derivatization with FBS, brown bands were observed both in the LxHs and the control plate of gallic acid (Figure 1B), which is in line with the results of Royer [38]; these bands could indicate the presence of tannins or phenolic compounds. The derivatization with NP/PEG and Dragendorff showed the absence of flavonoids and alkaloids in the extract, respectively (Figure 1C,D). In brief, these qualitative results indicate the occurrence of terpenes or steroids, tannins or phenolic compounds, and the absence of flavonoids and alkaloids in LxHs.

**Figure 2:**
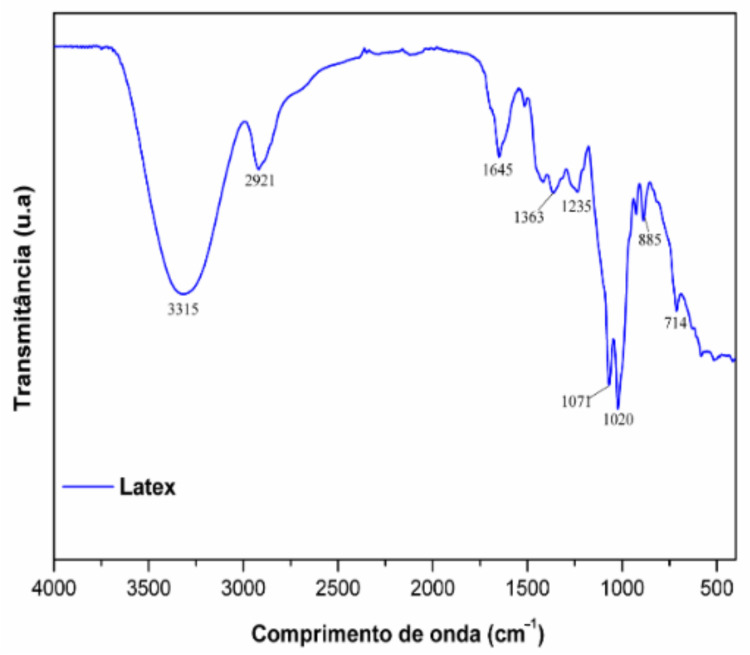
FT-IR from *Hancornia speciosa* Gomes (LxHs) aqueous extract.

In the FT-IR analysis (Figure 2), it was possible to observe aliphatic compounds evidenced by medium intensity bands at 2921 cm^−1^—characteristic of C-H bonds—and the absence of aromatic compounds’ typical absorptions. The intense stretch at 3315 cm^−1^ is attributed to O-H bonds; strong intensity bands evidenced the hydroxyls’ aliphatic nature at 1071 cm^−1^ and 1020 cm^−1^, representing C-O bonds in alcohols. Finally, the absorption at 1645 cm^−1^ is attributed to bonds between carbonyl and cyclic unsaturated α β systems [39,40,41].

**Figure 3:**
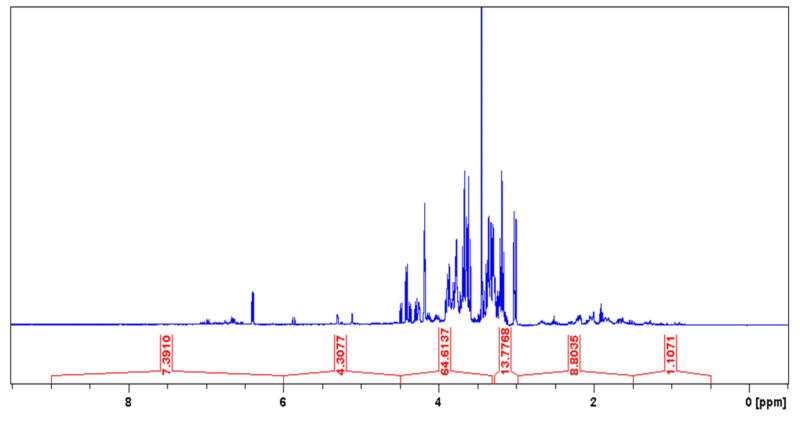
^1^H NMR spectra from the *Hancornia speciosa* Gomes (LxHs) aqueous extract.

In the ^1^H NMR assessment of LxHs, it was observed that the highest percent area (78.39%) was between δ values from 3.0 to 4.5 ppm, corresponding to hydrogens bound to hydrated carbons (CH_3_–OH/CH_2_–OH/CH–OH), alkoxy groups (PhO–CH_3_/PhO–CH_2_/RCOO–CH_3_/RCOO–CH_2_/RCOO–CH), or hydrogens adjacent to carbonyls (CH_3_–CO–R/CH_2_–CO–R/CH–CO–R/H–CO–OR). The area between 1.5 and 3.0 ppm was 8.8%, corresponding to hydrogens from –CH_3_/-CH_2_/-CH adjacent to unsaturated carbons (CH_3_–C=C/CH_2_–C=C/CH–C=C), ketone or aldehyde carbonyl (CH_3_–COR/CH_2_–COR/CH–COR), or carboxyl (CH_3_–COOR/CH_2_–COOR/CH–COOR) or directly bound to an aromatic ring (CH_3_–Ph/CH_2_–Ph/CH–Ph). It was also observed that there were hydrogens between 4.5 and 6.0 ppm with area equal to 4.31% corresponding to olefin hydrogens (CH_2_=CH–R/R–CH=CH–R) and between 6.0 and 9.5 ppm (7.39% area) corresponding to hydrogens bound to aromatic carbons (H-Ar) or olefin hydrogens adjacent to an aromatic ring (Ar–CH=CH–R). The lowest area (1.11%) was from 0.5 to 1.5 ppm, corresponding to alkyl hydrogens (–CH_3_, –CH_2_, –CH) (Table 1, Figure 3) [42,43,44].

**Figure 4:**
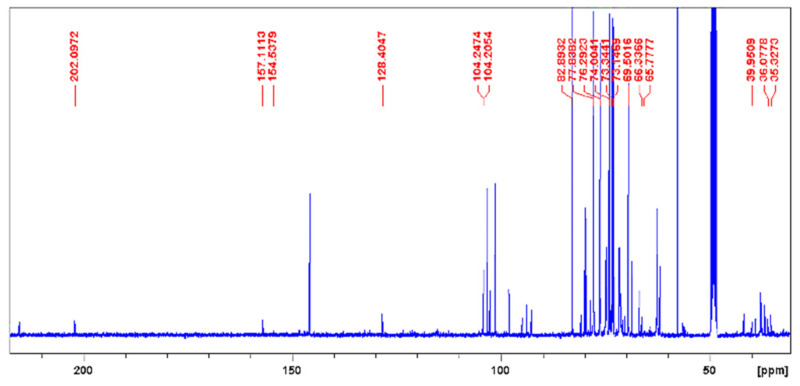
^13^C NMR spectra from the *Hancornia speciosa* Gomes (LxHs) aqueous extract.

The ^13^C NMR from the latex’s aqueous extract corroborated the presence of aliphatic carbons (–CH_3_, –CH_2_–, –CH–, CH_3_–CO–, –CH_2_–NH_2_) with δ values ranging from 10.0 to 50.0 ppm. The area between 50.0 and 90.0 ppm—typical of aliphatic carbons from ether and alcohols (–C–O–C–, –C–OH)—had the most intense signals, in line with the ^1^H NMR (δ signals from 3.0 to 4.5 ppm) and the FT-IR. Besides, the area of aromatic and olefin carbons (δ from 100.0 to 160.0) had the least intense signals, as also observed in the ^1^H NMR (δ from 4.5 to 6.0 ppm), as shown in Figure 4.

**Figure 5:**
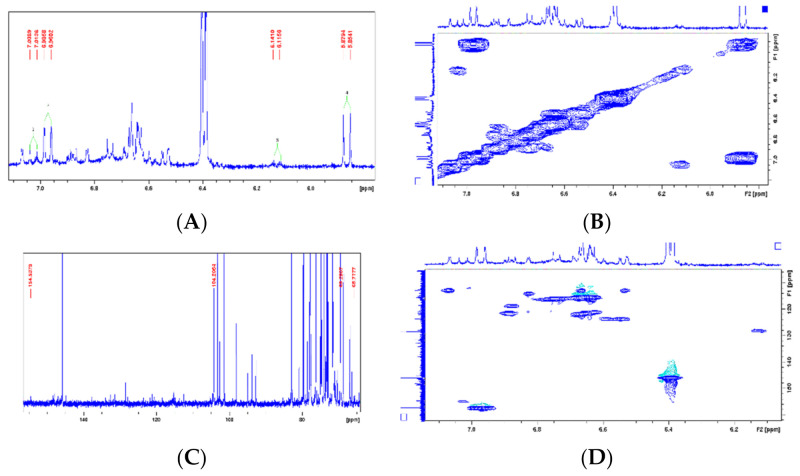
(**A**): ^1^H NMR spectra (Expansion 1) from the *Hancornia speciosa* Gomes (LxHs) aqueous extract. (**B**): 2D ^1^H-^1^H COSY NMR spectra correlation map spectra from the *Hancornia speciosa* Gomes (LxHs) aqueous extract. (**C**): ^13^C NMR spectra (Expansion 1) from the *Hancornia speciosa* Gomes (LxHs) aqueous extract. (**D**): 2D ^1^H-^13^C HSQC NMR spectra correlation map from the *Hancornia speciosa* Gomes (LxHs) aqueous extract.

Comparing the NMR data with the literature, we identified one structure as cornoside, a precursor of haleridone [13,45,46]. In the ^1^H NMR spectrum expansion (Figure 5A), two signal clusters were observed at 7.02 and 6.12 ppm; in this tangle of signals, four signals were identified with coupling constant of olefin hydrogens (J = 10.1 Hz), attributed to the hydrogens H-3/H-5 and H-2/H-6, of cornoside. The correlation between signals H-2 and H-3 and between H-5 and H-6 was confirmed by the COSY homonuclear 2D spectra (Figure 5B). As observed in the ^13^C NMR (expansion), there is a group of signals typical of unsaturated olefin carbons (C=C) at 177.8 and 154.5 ppm. The signal at 104.2 ppm is attributed to the anomeric carbon of glucose (Figure 5C). Collectively, these data indicate the presence of a glycosylated ethylcyclohexanoid, as the molecule cornoside [47,48]. The HSQC analysis could confirm the correlation between hydrogens H-3/H-5 (δ = 7.02 ppm) and H-2/H-6 (δ = 6,12 ppm) and carbons C-3/C-5 (δ = 154,5) and C-2/C-6 (δ = 154.5 ppm), respectively (Figure 5D).

Another compound was identified through NMR. The ^1^H NMR analysis (expansion) detected two signals from olefin hydrogens at 6.97 and 5.86 ppm (Figure 5A), whose coupling constants are typical of cis hydrogens (J = 10.2 Hz); these signals are commonly related to H-2 and H-3 from cyclohexylethanoid and were confirmed through 2D COSY (Figure 5B) due to the structural similarities. In the ^13^C NMR spectra, a group of signals typical of olefin unsaturated carbons (C=C) at 128.4 and 157.1 ppm; a carbon signal at 202 ppm typical of α,β-unsaturated ketone, confirmed by the olefin signals; and a signal at 104.2 ppm attributed to the anomeric carbon from glucose were further observed. Overall, these data indicate the presence of another glycosylated ethylcyclohexanoid (Figure 5C) [13,49]. An HSQC contour analysis could confirm correlation between hydrogens H-2 (δ = 6.97 ppm) and H-3 (δ = 5.86 ppm) and the carbons C-2 (δ = 157.1) and C-3 (δ = 128.4), respectively (Figure 5D). By assessing all signals and correlations (Table 2) and comparing the results with the literature [13,50], we identified such structure as the glycosylated ethylcyclohexanoid dihydrocornoside.

**Figure 6:**
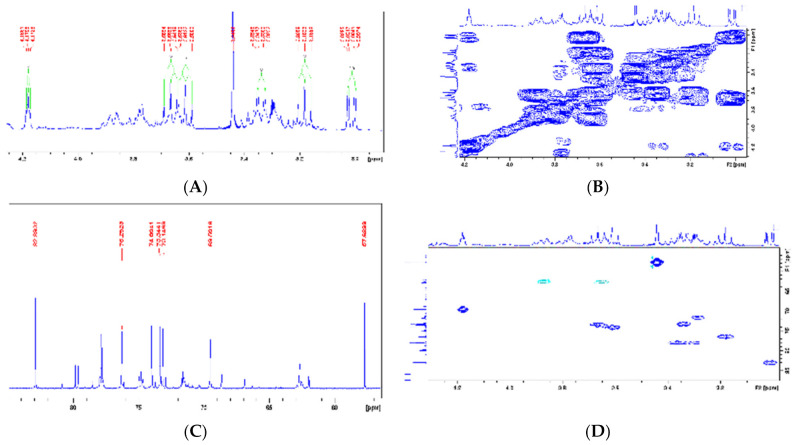
(**A**): ^1^H NMR spectra (Expansion 2) from the *Hancornia speciosa* Gomes (LxHs) aqueous extract. (**B**): 2D ^1^H-^1^H COSY NMR spectra (Expansion 1) correlation map from the *Hancornia speciosa* Gomes (LxHs) aqueous extract. (**C**): ^13^C NMR spectra (Expansion 3) from the *Hancornia speciosa* Gomes (LxHs) aqueous extract. (**D**): 2D ^1^H-^13^C HSQC NMR spectra (Expansion 2) correlation map from the *Hancornia speciosa* Gomes (LxHs) aqueous extract.

**Figure 7:**
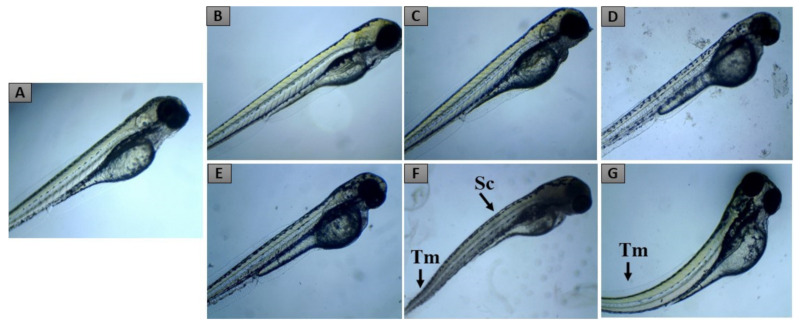
Representative pictures of zebrafish larvae from the groups CS (**A**), CD (**B**), C1 (**C**), C2 (**D**), C3 (**E**), C4 (**F**), and C5 (**G**), exposed through immersion to LxHs up to 96 hpf. In (**A**), (**B**), (**C**), (**D**), and (**E**), the larvae are under typical development, while in (**F**) (C4) and (**G**) (C5), some larvae show scoliosis (Sc) or tail malformation (Tm).

Moreover, ^1^H NMR analysis of the LxHs showed seven signals typical of hydroxyl-bound carbons (δ values from 3.0 to 4.0 ppm). Among these, an intense singlet was observed at 3.44 ppm corresponding to three hydrogens from methoxy groups (O-Me), and two doublets were observed at 3.01 and 3.33 ppm with coupling constants (J) 9.8 and 2.8 Hz, indicating the occurrence of two coupling systems Hax-Hax-Heq (trans–diaxial–geminal). A triplet signal was observed at 4.18 ppm with J = 3.0 Hz, characteristic of Hax-Heq-Hax, indicating single hydrogen at an equatorial position, attributed to H-2. A group of hydrogens with triplet signals at 3.18, 3.66, and 3.61 were observed, with J = 9.4 Hz, consistent with trans–axial scalar coupling Hax-Hax-Hax, which were attributed to H-5, H-6, and H-4, respectively. The values of trans–diaxial and axial–equatorial coupling constants observed in the sample are typical of cyclohexane systems, whose Jax-ax varies from 8 to 14 Hz and Jax-eq varies from 2 to 3 Hz [40,41]. These data indicate the chair conformation of cyclohexane, resulting in fewer hydroxyls at the axial position (Figure 6A) [51]. The hydrogen correspondence assessed through ^1^H NMR further was confirmed through 2D COSY (Figure 7B). The ^13^C NMR spectra from LxHs showed signals from oxidized methyl and methylidyne carbons at 57.6 ppm that was attributed to the methoxy group (OMe), and a carbon signal with higher chemical shift (δ = 82.9 ppm) is also observed, indicating methylation due to the inductive effect produced by this group (Figure 7C) [29,52]. The signal assignments were analyzed through 2D HSQC correlation map analysis showing that C-2 (δ = 69.5 ppm) was bound to H-2 at equatorial position (δ = 4.18 ppm), C-1 (δ = 82.9 ppm) was bound to the hydrogen at 3.33 ppm, C-4 (δ = 74 ppm) was bound to H-4 (δ = 3.61), C-5 (δ = 76.2 ppm) was bound to H-5 (δ = 3.18 ppm), and C-6 (δ = 73.3 ppm) was bound to H-6 (δ = 3.66 ppm), as shown in Figure 7D and Table 2. Comparing these data with the literature [53,54,55], we concluded that the sample contained the metabolite 1-O-methyl-myoinositol (bornesitol).

### 2.2. Zebrafish Embryo Acute Toxicity

The toxicity assessment on zebrafish embryos was performed by exposing them to different concentrations of LxHs and observing the frequency of embryo lethality and teratogenesis in different periods (Table 3). The results show that mortality and teratogenesis were observed only in the embryos exposed to the highest dose tested, with tail malformation and scoliosis occurrences (Figure 7). The only lethal alteration observed was coagulated eggs, and no lack of heartbeat was observed. It was impossible to calculate the LD_50_ with the concentrations assessed, evidencing the low toxicity of the extract.

### 2.3. Adult Zebrafish Acute Toxicity

**Figure 8:**
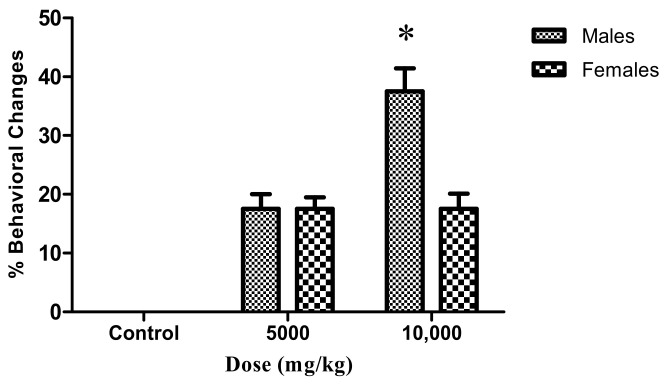
Effect of oral treatment with control (distilled water) and doses of LxHs at 5000 and 10,000 mg/kg on behavioral alteration in adult male and female zebrafish. The bars represent the mean ± SD; * *p* < 0.05 (ANOVA followed by Dunnett’s post hoc test).

Treatment of adult fishes with doses of LxHs of 5000 and 10,000 caused behavioral changes in a dose-dependent fashion. The most notable changes were observed in male fishes with the highest dose (Figure 8). Among these changes, there were stress signals such as tail tremors and rest at the tank bottom. However, all the animals recovered, and no death was observed, evidencing the low toxicity of the extract.

#### General Histopathology

**Figure 9:**
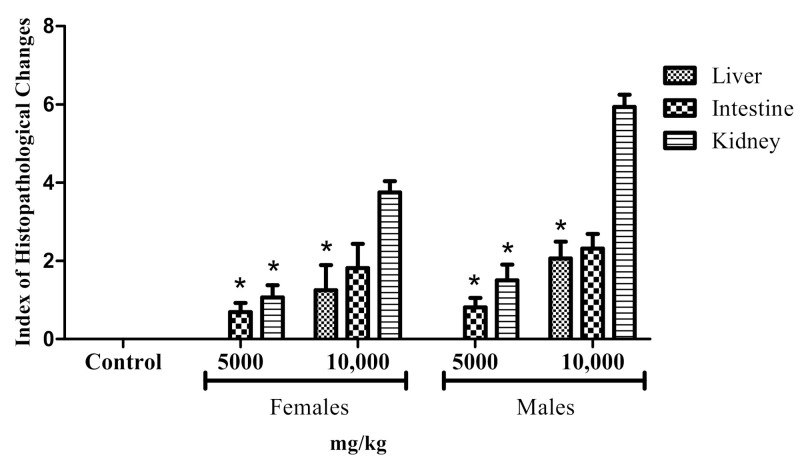
Index of histopathological changes. Data are presented as mean ± SD (*n* = 12/group); * *p* < 0.05 compared to the control (one-way ANOVA, followed by Dunnett’s post hoc test).

**Figure 10:**
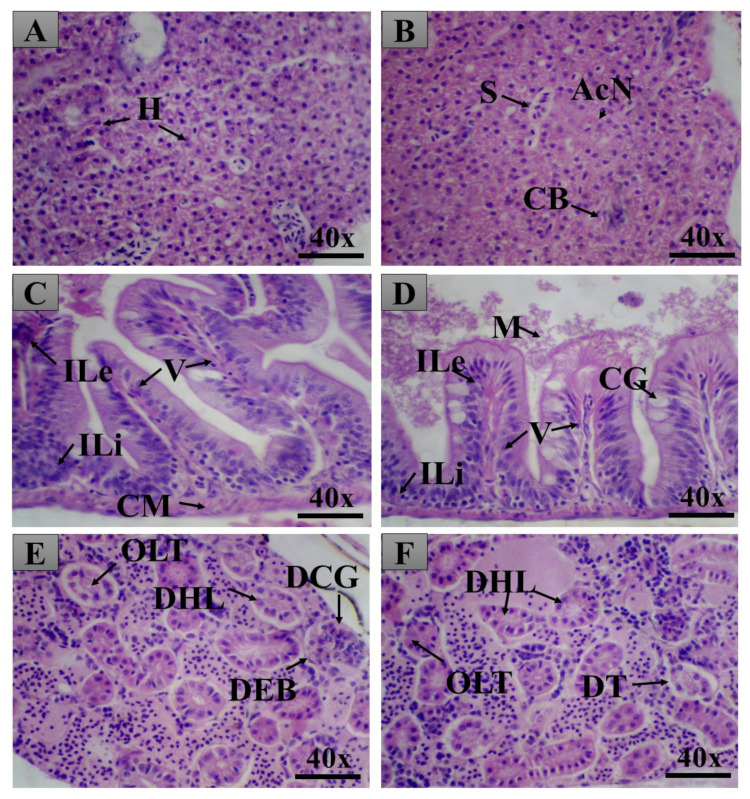
Pictures from the organs of female adult zebrafish. Top: liver; Middle: intestine; Bottom: kidney. Pictures on the left are from animals treated with LxHs at 5000 mg/kg; pictures on the right are from animals treated with LxHs at 10,000 mg/kg. In (**A**), it is possible to observe typical liver hepatocytes (H). In (**B**), it is possible to observe liver sinusoids (S), atypical contour of the nuclei (AcN), and canaliculi of the bile (CB). (**C**) shows the intestine with infiltration of leukocytes (ILe) and lymphocytes (ILi), villi (V), and the muscle layer (CM). (**D**) shows infiltration of leukocytes, lymphocytes, villi, goblet cells (CG), and mucus (M). In (**E**)**,** it is possible to observe obstruction of the kidney tubules (OLT), light hyaline degeneration (DHL), dilation of the glomeruli capillary (DCG), and decrease in the Bowman capsule space (DEB). Finally, (**F**) shows obstruction of the kidney tubules, light hyaline degeneration, and tubular degeneration (DT). H&E X 40.

**Figure 11:**
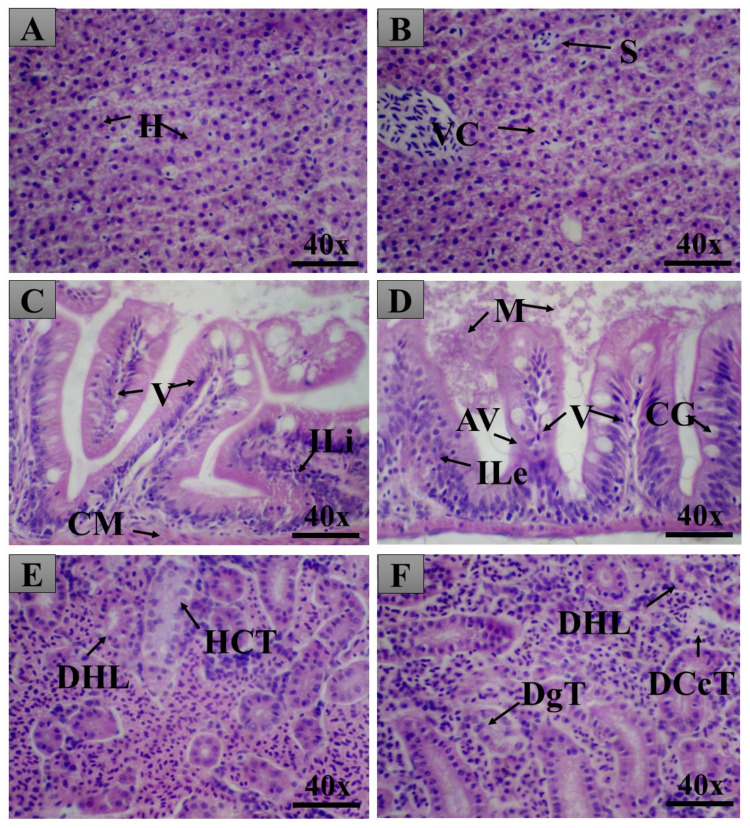
Pictures from the organs of male adult zebrafish. Top: liver; Middle: intestine; Bottom: kidney. Pictures on the left are from animals treated with LxHs at 5000 mg/kg; pictures on the right are from animals treated with LxHs at 10,000 mg/kg. In (**A**), it is possible to observe typical liver hepatocytes (H). In (**B**)**,** it is possible to observe hepatocytes with cytoplasmic vacuolization (VC). (**C**) shows the intestine with infiltration of lymphocytes (ILi), villi (V), and the muscle layer (CM). (**D**) shows the intestine with infiltration of leukocytes (ILe), villi, goblet cells (CG), mucus (M), and atrophy of the villi (AV). (**E**) shows the kidney with light hyaline degeneration (DHL) and hyperplasia of tubular cells (HCT). Finally, (**F**) shows the kidney with light hyaline degeneration, tubular degeneration (DgT), and cytoplasmic degeneration of tubular cells (DCcT). H&E X 40.

The oral treatment of the male and female adult animals produced few tissue changes in the liver, intestine, and kidneys, which were assessed by calculating the index of histopathological changes (Figure 9, Figure 10 and Figure 11). The LxHs at 5000 mg/kg did not cause tissue changes in the animals’ livers. At 10,000 mg/kg, a few tissue changes were observed in males (2.06 ± 0.427), more than were observed in females (1.25 ± 0.645). However, these values are considered low, and the tissues changes observed were of stage I only (Figure 11), including atypical nuclear contour and cytoplasmic vacuolization.

All the LxHs treatments caused tissue changes in the intestine, but the values are considered low (0.687 ± 0.239 for females and 0.812 ± 0.375 for males), and the organ was still inside the normal range (Figure 9). The tissue changes observed were leucocyte infiltration in the stroma and mucus between the lamellae (Figure 10 and Figure 11).

The highest degree of tissue alteration was observed in the kidneys; for 5000 mg/kg, the index was 1.06 ± 0.314 for females and 1.50 ± 0.408 for males. However, the indexes were higher for 10,000 mg/kg (3.75 ± 0.288 for females and 5.93 ± 0.314 for males). More frequently observed tissue changes were tubular lumen obstruction, light hyaline degeneration in the tubules, glomeruli capillary dilation, decreased Bowman capsule space, and tubular degeneration. Despite being the most affected organ, according to the index of histopathological changes, these values are still inside the normal range (between 0 and 10).

### 2.4. LxHs Antidiabetic Activity

**Figure 12:**
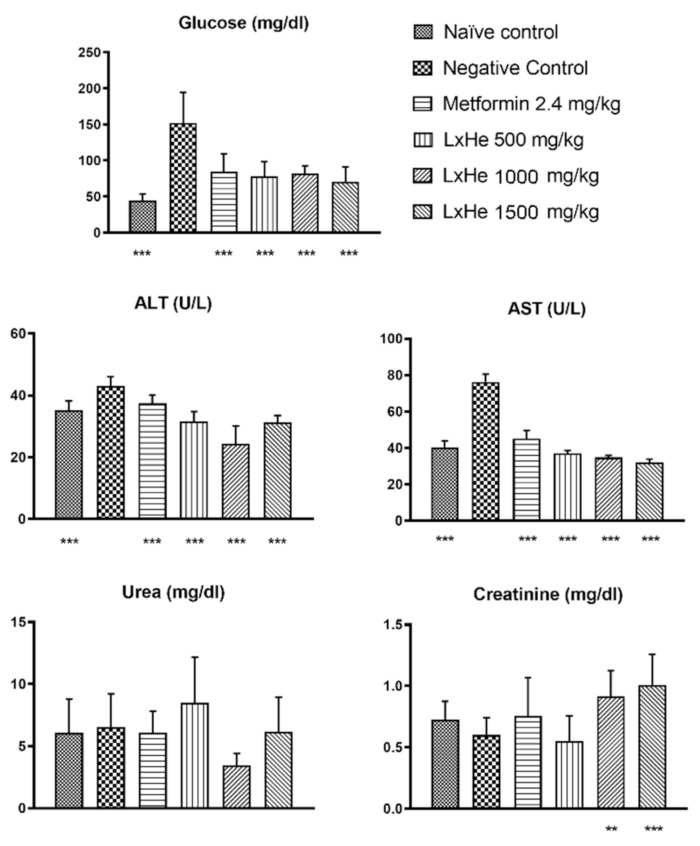
Effect of treatment (single dose) orally with *Hancornia speciosa* Gomes (LxHs 500, 1000, and 1500 mg/kg) in zebrafish. Results of glucose and other biochemical parameters in the animals after their respective treatments. The bars represent the mean ± SD; **: *p* < 0.01; ***: *p* < 0.001 (ANOVA followed by Dunnett’s post hoc test).

In this study, it was observed that all diabetic animals treated with LxHs had significantly decreased glycemic levels (*p* < 0.001; ANOVA with Dunnett’s post hoc test; Figure 12), which were similar to the group treated with metformin (84.5 ± 25.7 mg/dL). Their average glycemia values were 77.5 ± 20.2 mg/dL (500 mg/kg), 82.1 ± 10.7 mg/dL (1000 mg/kg), and 70.6 ± 20.4 mg/dL (1500 mg/kg), while the nondiabetic group’s glycemia was 44.8 ± 8.5 mg/dL. On the other hand, the negative control group, composed of diabetic animals treated only with water, had an average of 152.4 ± 41.8 mg/dL.

### 2.5. Effects of the Treatments on Biochemical Parameters

Hepatotoxicity is a critical factor in the development of novel drugs. A common way to evaluate it is through the activity of the enzymes alanine transaminase (ALT) and aspartate transaminase (AST); this is often performed in zebrafish as well [56,57]. While highly increased ALT activity can be a signal of liver injury due to cell membrane damage [58], highly increased AST activity is considered an indicator of mitochondrial damage [58,59].

Only the negative control group had abnormal ALT values (43.1 ± 2.85 U/L). According to Kitamura [60] and Huang [61], typical ALT values are between 5 and 35 U/L in teleosts. All groups treated with LxHs had average values within the healthy range and were statistically different from the negative control group (*p* < 0.001; ANOVA with Dunnett’s post hoc test; Figure 12), and the lowest values were observed with 1000 and 1500 mg/kg (24.2 ± 5.88, 31.3 ± 2.17 U/L). Similarly, all groups had statistically lower AST activity values compared to the negative control group, and a dose-dependent decrease in AST activity was observed in the groups treated with the extract (36.9 ± 1.62, 34.8 ± 1.06, and 32.2 ± 1.64 U/L for 500, 1000, and 1500 mg/kg, respectively).

### 2.6. Pancreas Histopathology

**Figure 13:**
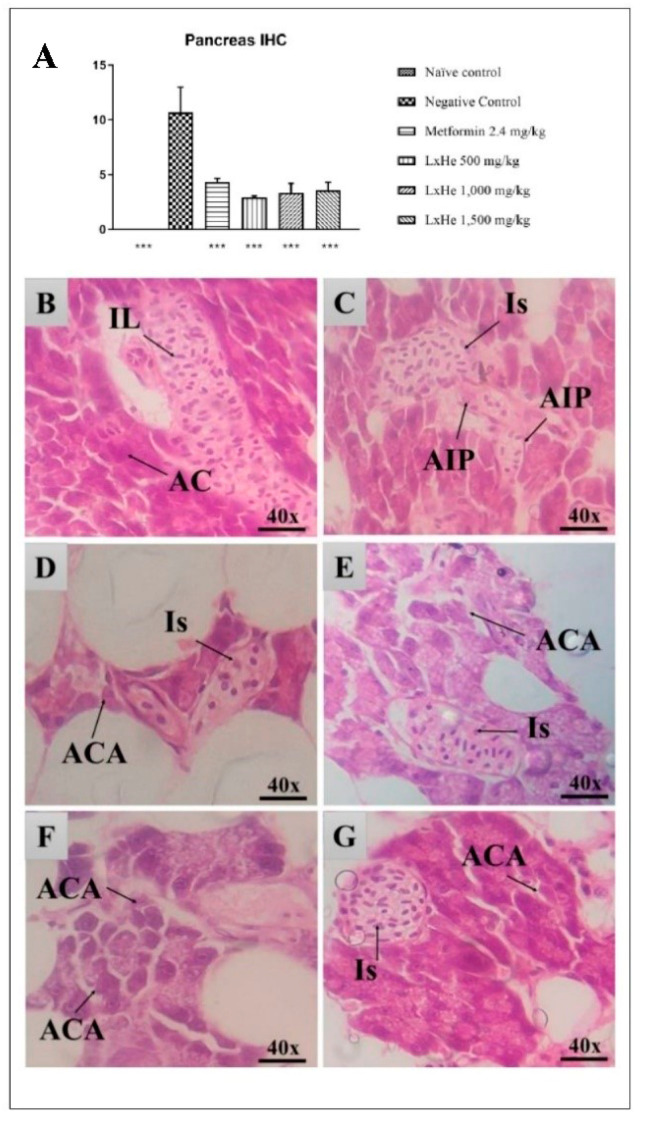
(**A**) shows the effect of oral treatment (single dose) with *Hancornia speciosa* Gomes (LxHs 500, 1000, and 1500 mg/kg) on index of histopathological changes for pancreas in zebrafish. The bars represent the mean ± SD; ***: *p* < 0.001 (ANOVA followed by Dunnett’s post hoc test). (**B**–**G**) show representative pancreas’ slides from animals treated with LxHs or controls. B: pancreas of a naïve control animal in its typical aspects with islets of Langerhans (IL) and acinar cells (CA); (**C**): pancreas of a diabetic animal that received only water, where insulitis (Is) and atrophy of the pancreatic islets (AIP) are observed; (**D**): pancreas of a diabetic animal treated with metformin at 2.4 mg/kg, where insulitis (Is) is observed; (**E**–**G**): pancreas from diabetic animals treated with LxHs at 500, 1000, and 1500 mg/kg, where atrophy of acinar cells (ACA) and insulitis (Is) can be observed. Although some tissue changes can be observed in animals treated with metformin and LxHs, the animals’ organs were healthy, as evidenced by the index of histopathological changes <10. H&E ×40.

In this study, we also observed the occurrence of pyknotic nuclei in the pancreas of diabetic animals. The diabetic animals treated with metformin and the three doses of the extract had very few tissue changes in the pancreas. We calculated the index of histopathological changes (IHC) through a systematic analysis of tissue changes, shown in Figure 13A. No tissue changes were observed in the naïve group in which the diabetes was not induced (Figure 13A). On the other hand, diabetic animals that received only water had mild to moderate tissue changes (IHC = 10.66 ± 2.33). The most common stage I tissue changes observed in this group were pyknotic nuclei and the presence of lymphocytes and monocytes. The most common stage II tissue changes were insulitis and islet atrophy. No stage III tissue change was observed.

There was a statistical difference between the groups treated with metformin or extract and the negative control group (*p* < 0.001; ANOVA with Dunnett’s post hoc test). All these groups had IHC values lower than 10, which means normal function (metformin 2.4 mg/kg: 4.33 ± 0.33; LxHs 500 mg/kg: 2.91 ± 0.14; LxHs 1000 mg/kg: 3.36 ± 0.84; LxHs 1500 mg/kg: 3.58 ± 0.72). The most common stage I tissue changes found in these groups were the presence of lymphocytes, monocytes, and acinar cell atrophy (Figure 13B–G). As for stage II, the most common change was insulitis, and no stage III tissue change was observed.

Collectively, the histopathology and biochemical parameters show that all doses were secure and did not induce any form of toxicity, in line with the acute toxicity assessment. This is in accordance with [24], a study reporting that no toxicity was observed with doses up to 100 mg/kg and no deaths occurred with doses up to 1.5 mg/kg.

### 2.7. In Silico Results

The outputs given by the server between inositol and receptors involved in the regulation of glycemia are shown in Table 4. The enzymes with E-values closer to zero were α-galactosidase, α-fucosidase, glucosylceramide, and β-galactosidase. Contrarily, MaxTc values were reasonably low, ranging from 0.29 to 0.45. Based on the SEA results and the crystallographic structures’ availability with an appropriate resolution, we performed a docking of inositol with the enzymes galactosidase and maltase-glucoamylase.

**Figure 14:**
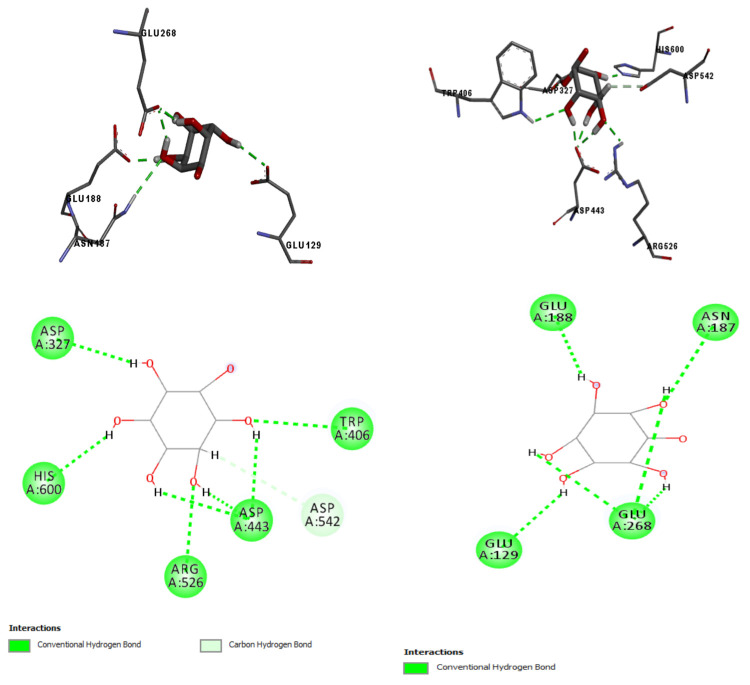
Molecular docking poses calculated by GOLD between inositol and maltase-glucoamylase (**left**; PDB ID: 2QMJ) and β-galactosidase (**right**; PDB ID: 3THC). (**Top**): 3D representation; (**Bottom**): 2D representation.

Molecular docking is a powerful tool used in drug discovery to simulate an interaction profile between the studied ligand and the receptor’s active site [62]. There were eight interactions between inositol and six amino acids from maltase-glucoamylase’s active site (ASP327, TRP406, ASP443, ARG526, ASP542, and HIS600; Figure 14), and the fitting score (Goldscore) was 48.84. Inositol had only one dipole–dipole interaction (ASP542), and all others were conventional hydrogen interactions. According to Sim [63], the amino acid ASP443 of maltase-glucoamylase behaves as a catalytic nucleophile while ASP542 behaves as an acid–base catalyst. Both of them interacted with inositol, but the latter formed more hydrogen interactions.

Maltase-glucoamylase has an N-terminal active site and a C-terminal active site. The docking showed more favorable interactions toward the N-terminal site. This can be explained by the fact that this active site is more prone to interacting with low-weight molecules due to the small size of its catalytic site. In fact, other small molecules used to treat diabetes, such as miglitol and voglibose, have a higher inhibition potential in the N-terminal site than acarbose [64].

β-galactosidase is another enzyme involved in carbohydrate metabolism whose inhibition reduces catalysis of larger carbohydrates, reducing the formation of glucose and other monosaccharides [65]. β-galactosidase is also known as lactase because it is accountable for breaking lactose into glucose and galactose. Inositol had a GoldScore equal to 46.77 toward this enzyme; there were six hydrogen interactions with the amino acids ASN187, GLU188, GLU129, and GLU268. According to Ohto [66], the amino acids GLU268 and GLU188 act as a catalytic nucleophile and an acid–base catalyst, respectively. The docking showed inositol could interact with both of them, mainly with GLU268, forming interactions with distances lower than 2.2 Å. The strong interaction between inositol molecules could hamper its catalytical activity of forming glucose and other monosaccharides that would be absorbed into the blood. This could be a plausible mechanism in which the extract exerted its hypoglycemic effect since the phytochemical study pointed to the presence of bornesitol.

## 3. Discussion

In brief, the phytochemical analysis indicates that the latex’s aqueous extract has the molecules cornoside, dihydrocornoside, and 1-O-methyl-myoinositol (bornesitol)—a cyclitol from the group of inositols [48,50]. Inositols are ubiquitous polyols with several physiological roles. They are produced endogenously and can be found in several foods and dietary supplements. Alterations in absorption, metabolism, or excretion of inositols seem to have an important role in metabolic diseases involving insulin resistance. Recently, inositol has been gaining attention in the treatment of such diseases [67]. However, other molecules can be present in the extract since the HPTLC and derivatization suggested the occurrence of terpenes or steroids and tannins or phenolic compounds. After assessing the chemical composition of LxHs, we performed the in vivo studies of LxHs treatment on an in vivo model of diabetes in zebrafish.

This study used a chemically induced model of diabetes caused by the death of pancreatic beta cells by alloxan. These cells are accountable for producing insulin, and hence, a metabolic disturbance occurs due to increased glycemic levels and reduced insulin levels, similar to diabetes mellitus [68,69].

The zebrafish has gained attention not only in the study of diabetes but also in the study of a range of other metabolic diseases [70]; this is possible because the animal’s glucose metabolism is very similar to that of mammals [71,72,73]. Under typical conditions, the glucose level of zebrafish is around 60 mg/dL [74] and is dynamically regulated according to its feeding [75]. Zang [76] reported that after seven days of metformin treatment in diabetic animals, the blood glucose was significantly reduced compared to nontreated animals, just as observed in our study. In this sense, metformin acts as an adequate control antidiabetic drug, improving the model’s validity.

One study reported that the leaves of *H. speciosa* Gomes exerted antidiabetic activity [24]. The authors reported that the extract and all fractions tested could inhibit the activity of α-glucosidase in vitro, but only the crude extract and dichloromethane fractions inhibited hyperglycemia caused by glucose and starch in mice. Moreover, both of them increased glucose uptake into adipocytes. The extract had in its composition bornesitol, quinic acid, chlorogenic acid, and flavonoid glycosides. The authors mention that this could be due to cyclitols and flavonoids since these molecules can decrease glycemic levels by increasing glucose uptake. Although the study was performed using leaf extracts, some compounds were observed in LxHs, such as the cyclitol bornesitol.

Marinho [14] reported that the aqueous extract exerted anti-inflammatory and antinociceptive activity in mice using several models, corroborating its traditional use as an anti-inflammatory agent. The treatment decreased the nociceptive action of formalin in the second phase (inflammatory phase), decreased the carrageenan-induced edema at all time points, decreased exudate volume and protein concentration in the air pocket model, decreased the activity of iNOS and COX-2, and decreased the levels of the inflammatory mediators TNF-α and IL-6. Such anti-inflammatory activity is relevant and can be involved in latex’s hypoglycemic activity because there is a close link between inflammation and diabetes, as inflammatory cytokines can increase insulin resistance.

In accordance with these results, it was observed here that the animals treated with different concentrations of LxHs had significantly lower glucose levels than the untreated group.

The majority of animals produce some urea, but little is known about the factors affecting its metabolism in teleosts [77]. However, according to Thrall [78], the levels cannot be higher than 10 mg/dL. In our study, the group treated with the lower dose of the extract had the highest value (8.50 ± 3.65 mg/dL) but was still within normality, and the lowest value was observed with the dose of 1000 mg/kg (3.43 ± 0.97 mg/dL). Overall there were no statistical differences compared to the negative control group (Figure 12). Typical creatinine levels are between 0.5 and 2 mg/dL in teleosts [78]. We observed a significant difference in creatinine values in 1000 mg/kg and 1500 mg/kg compared to the negative control group (*p* < 0.01 and *p* < 0.001; ANOVA with Dunnett’s post hoc test; Figure 12); however, all groups were within the normality range.

The pancreas of zebrafish has the same function as the other vertebrates [79]; as in other teleosts, it is a diffuse organ spread around the intestine, as little globules in the fish mesentery. It works as an endocrine organ by secreting insulin and glucagon and as an exocrine organ by secreting pancreatic juice [80,81]. The endocrine function is performed by islets of Langerhans that consist of agglomerates of glandular, light-colored cells. The most important products of these islets are glucagon and insulin, which are produced by α and β, respectively [79,82]. Benchoula [83] reported that zebrafish treated with alloxan had considerably lower cellularity than healthy animals; this is in accordance with what was observed in our study.

Diabetes mellitus induction leads to decreased number and size of the islets, leucocyte infiltration, and β cell degranulation, caused by insulin depletion; it can decrease cell mass as well [84]. In teleosts, degranulation is also observed, along with nuclear hypertrophy; the cells may have an abnormal shape and be filled with glycogen. In more severe conditions, flaws can be observed in the cytoplasm with pyknotic nuclei [85].

**Figure 15:**
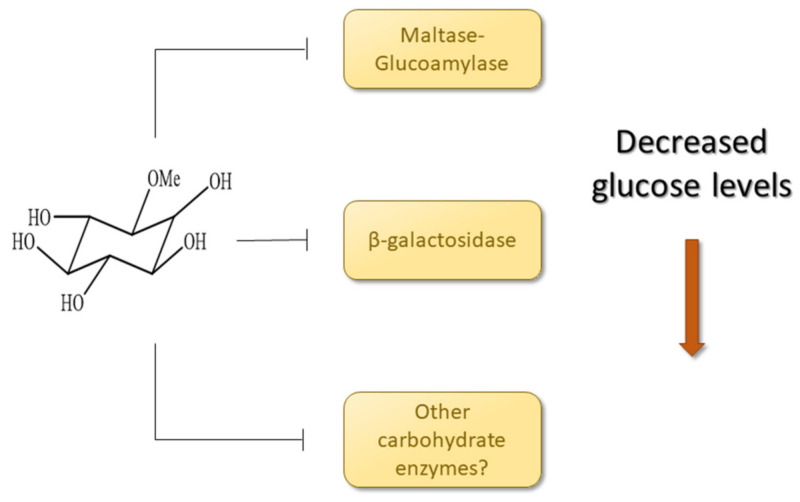
Schematic diagram showing some of the mechanisms in which the extract exerts its hypoglycemic effect based on the in silico results.

Next, we sought to understand how the extract would work through in silico studies. The SEA server provides an activity prediction to researchers based on the similarity of the molecule with groups of compounds that interact with a particular receptor, all in its database. The server output is given as an E-value and max Tanimoto coefficient. E-values are considered satisfactory when lower than 1 × 10^−10^, and the closer the E-value is to zero, the higher the similarity is toward the group of compounds that interact with the given receptor. The Tanimoto coefficient varies from 0 to 1, where 0 means total dissimilarity and 1 means identity; the server gives the max Tanimoto coefficient (MacTc) between the ligand and the group of compounds of its database that interact with the receptor assessed [86]. After assessing the molecule in the SEA server for interaction with carbohydrate enzymes, we sought to perform a docking simulation with inositol with maltase-glucoamylase and β-galactosidase. Due to the lack of enzymes in sufficient resolution to perform the docking, only these two were assessed, and hence it is possible that other enzymes are involved in the action of cyclitols, including bornesitol (Figure 15). Further studies are necessary to corroborate such mechanisms in vitro. We suggest that such antidiabetic activity is due to 1-O-methyl-myoinositol (bornesitol) in the extract since it is an inositol molecule whose class of compounds are known hypoglycemic agents. Nevertheless, other mechanisms should not be ruled out.

After assessing the composition, efficacy as a hypoglycemic agent, and a possible mechanism of action, we sought to evaluate the extract’s safety on acute toxicity models using embryos and adult zebrafish. In the embryos, the frequency of lethality and malformations were assessed. Only the highest extract concentrations could induce malformations such as tail malformation and scoliosis (91.05 mg/mL and 113.80 mg/mL). Notably, even the highest doses could not induce heart malformation in the embryos; this organ is the first to be formed in zebrafish and hence is essential to evaluate the toxicity in the embryos [87]. According to Mu [88], high concentrations of nocive compounds can change the heartbeat rate and cause edema, which was not observed with LxHs. According to Wang et al. [89], tail malformation and scoliosis can be assessed for teratogenic activity. He et al. [90] stated that tail malformation could be due to abnormal skeletal development. Here, these malformations were observed with the highest doses. However, even in the highest doses, their occurrence was rare considering the total number of embryos assessed (5%). Although some lethality was observed with the embryos, the amount of death was insufficient to calculate the LD_50_.

In the adults treated with LxHs at 5000 and 10,000 mg/kg, some behavioral changes were observed, mainly increased swimming. This was also observed by Souza et al. [16], evaluating the toxicity of *Acmella oleracea* extract. The behavioral changes start with increased swimming activity, which is a mechanism of defense to reduce the probability of death [15,78,91]. Other parameters evaluated could be body weight changes, among others [84], although not all of them are always assessed. Here, no death was observed in the adults treated with doses up to 10,000 mg/kg.

We then sought to look for signs of internal toxicity through histopathological analysis. This analysis can detect organ-specific toxicities [15,16,17,33,68]. According to Carvalho et al. [32], the liver of zebrafish is functionally similar to those of mammals, despite the structural divergences. The similarities include the pathways of drug metabolism, bile synthesis, and lipid and glycogen storage [16,17,92]. After exposure to nocive compounds, zebrafish liver histopathology can be compared to that of mammals due to its conserved physiology [33,93,94]. The results show that the tissue changes observed in this organ were low, not affecting its normal function. The cytoplasmic vacuolization observed in the animals treated with the extract at 10,000 mg/kg is very frequently reported in the literature [16,17,31,32,33] and is associated with decreased glycogen storage in the hepatocytes or lipid accumulation. In this study, however, the tissue changes were still within the normal range.

The intestine is vital to assess since it is the first organ affected by orally given compounds. In zebrafish, the intestine is formed by a mucous layer with goblet cells, inflammatory cells, and enterocytes [32]. This organ is also a place of enzyme and macronutrient recycling [95,96]. The observations assessed were according to Takashima and Hibiya [97]. The sensitivity of zebrafish intestine to nocive compounds has been determined through the assessment of tissue alterations [32,90]. We observed leukocyte and lymphocyte infiltration and the presence of mucous. These changes were also observed in [16,33], where the authors tested the toxicity of the nanoemulsion of *Rosmarinus officinalis* essential oil and *Acmella oleracea* extract, respectively. This tissue alteration could be due to inflammation of the lamina propria. According to the index of histopathological alterations, the tissue changes observed were not sufficient to compromise the organ function.

The kidney of zebrafish has nephrons responsible for filtrating blood residues and maintenance of the osmotic balance. The nephrons are composed of a renal corpuscle and proximal and distal convoluted tubules [16]. This organ, accountable for filtrating toxic compounds, is one of the most affected organs by them [32,82,97]. In accordance, it was the most affected tissue in our study. One of the tissue changes observed in this organ was tubular hyaline degeneration, an increased quantity of eosinophilic granules in the cytoplasm of these cells. This tissue change occurs due to the reabsorption of protein excess synthesized in the glomeruli [35]. The dilation of glomeruli capillary and decrease in Bowman capsule space are tissue changes that can hamper the function of the organ because the capillary dilation can decrease the space of the Bowman capsule, hindering the circulation and filtration of blood [31]. Here, this tissue alteration was not observed to a significant extent. According to [32], the tubular alterations in the kidney of zebrafish could be indirectly caused due to metabolic dysfunctions caused by toxic compounds. These alterations can eventually induce kidney necrosis if the toxicity is high enough [97]. In this study, the tubular changes, tubular degeneration, and hyperplasia of tubular cells were mild, and no necrosis or tissue dysfunction was observed.

Collectively, the acute toxicity tests indicate that the extract was secure in the embryos and adults up to high doses, and the tissue changes were not enough to compromise the organs. As a limitation, we acknowledge that more sensitive tests can be relevant, including ROS or apoptosis test in tissue sections, to evaluate the cellular response.

## 4. Materials and Methods

### 4.1. Plant Material

The plant material was collected in a private property in Macapá, Amapá, Brazil, under the coordinates 00 33′05.51160″ S, 50 50′30.14520″ W. The botanical identification was performed in the Science and Technology Research Institute of Amapá (IEPA) by Dr. Patrick de Castro Cantuária, and a dried plant specimen was stored under No. HAMAB 019176.

### 4.2. Hancornia Speciosa Latex and Its Aqueous Extract (LxHs)

The latex was collected traditionally, between 5 and 7 a.m., with a vertical cut in the stem with a machete [98]. The product was stored in dark vials with distilled water at 40 °C in 1:1 proportion over three hours to keep its consistency. Next, it was stored in a freezer at −10 °C to maintain its chemical integrity. The water-soluble part was extracted by defrosting the sample.

Aliquots of the aqueous latex extract (20 mL) were removed and dried in a greenhouse with a forced-air heater, resulting in 85 mg of mass. Then, we added 5 mL of methanol and put it under an ultrasonic bath for over 10 min. The insoluble part was removed, and the supernatant was filtered with a membrane filter (0.45 μm), yielding 65 mg of the extract. Finally, a clean-up was performed with 20 mg of the extract solubilized in a mixture of water and acetonitrile (2:8).

### 4.3. Phytochemistry

All solvents used were of analytical grade. Acetonitrile (ACN) and methanol (MeOH) were purchased from Tedia Company (Fairfield, CT, USA), and the ultrapure water was obtained through a Millipore Direct-Q3 system (18.2 MΩ.cm; Bedford, MA, USA). The ultrasonic bath was performed through direct contact using a Branson 2510 (Danbury, CT, USA), with frequency, potency, and temperature set at 42 kHz, 100 W, and 27 °C, respectively. The ^1^H and ^13^C nuclear magnetic resonance (NMR), homonuclear correlation spectroscopy (HOMO-COSY), and heteronuclear single quantum coherence (HSQC) spectra were obtained using a Bruker spectrometer Ascend model (Rheinstetten, Germany) in the range 400–100 MHz; the data were processed using the software TopSpin 3.6.0, and the FIDs were subjected to Fourier transform (LB = 0.3 Hz). The H_2_O resignal signal was suppressed by using presaturation sequences with selective low-potency irradiation. The spectra were manually processed, corrected at the baseline, and calibrated using as internal reference the residual nondeuterated fraction of the solvent CH_3_OH, centered on δ = 3.3 ppm [44,99,100,101,102]. The peaks were marked using the chemical displacement (δ) and coupling constants (J) of the unidimensional spectra ^1^H, ^13^C, homonuclear, and heteronuclear correlation maps (HOMO-COSY and HSQC).

The HPTLC was performed through an automated system composed of modules of application (Automatic TLC Sample 4), elution (Automated Multiple Development AMD 2), densitometer (TLC Scanner 4), and photo documentation (TLC Visualizer); all from the brand Camag (Muttenz, Switzerland). The stationary phase was composed of silica gel plates F-254 60 Å with glass support (Silicycle, QC, Canada). The mobile phase used was HPLC-grade (Tedia Company; Fairfield, USA) in gradient mode. The data were processed using the software WinCats 1.4.6. The automatic sprayer and thermal plate were from Camag (Muttenz, Switzerland). The analytical-grade reagents used for derivatization were vanillin (Nuclear), fast blue B salt (Merck), Dragendorff (Sigma, São Paulo, Brazil), NP/PEG (Sigma, São Paulo, Brazil), and potassium hydroxide (Nuclear, São Paulo, Brazil). Infrared (IR) spectra were obtained using a Bruker Vertex model (70 V) from 4000 to 400 cm^−1^, with 4 cm^−1^ resolution and 32 scans.

### 4.4. Samples Preparation and Analysis

For the HPTLC analysis, we prepared an LxHs solution at 5000 ppm in MeOH; 15 μL aliquots were injected into the plates with the standard solutions and then eluted through an isocratic system DCM/MeOH/Hfo (97:2:1). After being eluted and dried, the chromatographical separations were assessed under 254 nm and 366 nm wavelength radiation. Next, derivatization was performed within the chromatoplaques using the following solutions: NP/PEG for flavonoids, potassium hydroxide for coumarins and anthracene derivatives, 10% vanillin in sulfuric acid (VAS) for terpenes and acids, fast blue B salts (FBS) for tannins and phenolic compounds, and Dragendorff for alkaloids. These specific derivatizers were used in the plates with standard solutions of rutin, esculin, β-amyrin, gallic acid, and brucine, respectively. After the reactions, the NP/PEG and potassium hydroxide plates were exposed again to 366 nm wavelength radiation, while VAS, FBS, and Dragendorff were exposed to white light.

To obtain the 1D and 2D NMR spectra, 20 mg of the extract was solubilized in 600 μL of deuterated methanol (CD3OD). The latex’s aqueous extract was further evaluated through infrared spectroscopy with Fourier transform (FT-IR) using potassium bromide (KBr).

### 4.5. Animals

This study used AB wild-type adult zebrafish (Danio rerio) aged between 8 months and 2 years, weighing around 550 mg. The animals were purchased from the company Acqua New Aquarium and Fish Ltda. (Igarassu-PE, Brazil). All animals were kept under quarantine after arrival and were maintained in the Zebrafish Platform of the Drugs Research Laboratory, Biological and Health Sciences Department, Federal University of Amapá (UNIFAP), Brazil. The animals were kept in water under controlled temperature, feed, and light/dark cycle conditions, as described in the literature [31,33]. The Ethics Committee in Animals Use (CEUA) of UNIFAP approved this study under protocol No. 030/2018.

### 4.6. Embryos Acute Toxicity Assessment

The zebrafish embryos were treated with LxHs through immersion at the concentrations C1, 22.76 mg/mL; C2, 45.52 mg/mL; C3, 68.28 mg/mL; C4, 91.05 mg/mL; and C5, 113.80 mg/mL, diluted in system water. The control group was exposed to system water only (CS) and distilled water (CD). The embryos were collected through natural spam in reproduction tanks (Tecniplast). The collected eggs were washed and separated in plastic 92 mm Petri dishes (60 eggs per dish). The water temperature in the Petri dishes was kept at 26 ± 1 °C (50 mL).

The eggs were selected through examination with a stereomicroscope (Olimpo, Japan). Fertilized eggs without cleavage changes or chorion damage were selected. The selected fertilized eggs were transferred to a 96-well plate (20 embryos x 3 replicates) filled with 3 mL of their respective solution concentration. The embryo lethality features analyzed were egg coagulation, lack of somite formation, lack of tail displacement, and lack of heartbeats (24, 48, 72, and 96 hpf); positive result on any of these features means embryo death. Moreover, teratogenesis parameters were evaluated, including yolk edema, growth retardation (24, 48, 72, and 96 hpf), tail malformation, cardiac edema (48, 72, 96, and 120 hpf), and scoliosis (72 and 96 hpf) (Table 5)

### 4.7. Adult Toxicity Assessment

The adult animals, separated by sex, were treated with doses of 5000 and 10,000 mg/kg of the extract; in total, there were four groups with 12 animals in each. The animals were immobilized with a damp sponge and treated with LxHs with a micropipette (HTL Lab Solutions) using a maximum volume of 1.5 μL per animal [32,33,35]. Behavioral alterations and mortality were observed over 96 h.

The animals’ behavior was classified into three stages: (1) increased swimming activity, spasms, and tremors in the tail axis; (2) circular movement and loss of posture; (3) clonus, motility loss, immobility at the bottom of the tank, and death. Each animal was evaluated individually and was considered dead with a lack of response to mechanical stimulation and lack of operculum movement [31]. At the end of experiments, the animals were subjected to euthanasia through anesthetic cooling, according to the recommendation of the American Guidelines of the Veterinary Medical Association for Animal Euthanasia [103].

### 4.8. Diabetes Induction and Experimental Design

Each animal received three alloxan intraperitoneal (i.p.) injections: the first, at the beginning; the second, four days after; and the third, five days after the second according to the methodology described by Cueva-Quiroz, with adaptations [104]. The oral treatments with LxHs at 500, 1000, and 1500 mg/kg and metformin at 2.4 mg/kg began 24 h after the last alloxan injection and were performed over seven days, as described by Carvalho [32].

The groups (*n* = 16 animals per group) were divided as follows:

Group 1: Naïve control, nondiabetic (normoglycemic), without treatment;
Group 2: Negative control, diabetic, treated only with water (alloxan i.p. and water oral);
Group 3: Positive control, diabetic, treated with 2.4 mg/kg metformin (alloxan i.p. and metformin oral);
Group 4: Diabetic animal treated with LxHs 500 mg/kg (alloxan i.p. and LxHs oral);
Group 5: Diabetic animal treated with LxHs 1000 mg/kg (alloxan i.p. and LxHs oral);
Group 6: Diabetic animal treated with LxHs 1500 mg/kg (alloxan i.p. and LxHs oral).

### 4.9. Blood Collection and Biochemical Analyses

The blood collection and glucose measurement were conducted in animals after 10 and 12 h of fasting. Euthanasia was performed through rapid cooling between 0 and 4 °C until complete loss of opercular movement [105]. We did not use anesthesia drugs since they can induce altered glucose levels [106]. After euthanization, the animals were dried using a paper towel, then put on Petri dishes to remove 5 μL of blood from the tail. The glucose levels were measured with test strips and an On Call Plus (São Paulo, Brazil). The device can detect glucose levels in the range between 20 and 600 mg/dL.

Next, the plasma was separated by adding heparin to assess the levels of urea, creatinine, aspartate transaminase (AST), and alanine transaminase (ALT). Urea was assessed through UV photometry using the two-point/fixed-time kinetic method, and creatinine was assessed through colorimetry using a semiautomated biochemistry analyzer (Bioplus, BIO-200). AST and ALT were assessed through the UV kinetic method. Five microliters of blood was used for each analysis.

### 4.10. Histopathology Analysis

After euthanization, the animals were fixed in Bouin for 24 h to prepare the liver, intestine, kidney, and pancreas slides. After being fixed, the samples were decalcified in EDTA (Sigma Co., São Paulo, Brazil) for more than 24 h and dehydrated in a crescent concentration of ethanol (70%, 80%, 90%, and 100%). Next, they were diaphonized with xylene, embedded in paraffin, and sectioned in 5 µm slices using a rotary microtome (CUT: 6062, Slee Medical, Germany). Finally, the slides were stained with hematoxylin and eosin [31]. The images were assessed using an Olympus Microscope BX41 and photographed with a digital camera MDCE-5C USB 2.0.

The pancreas and other organs were assessed by calculating the index of histopathological changes (IHC). To calculate this index, the organ is observed to assess tissue changes classified according to its severity into stages I, II, and III (Table 6). IHC values from 0 to 10 indicate a typical organ, values between 11 and 20 indicate moderate tissue changes, values between 21 and 50 indicate moderate to severe changes, and higher values indicate severe irreversible changes [35,107]. The IHC is calculated according to the following equation:

$$I=\frac{\sum_{i-1}^{na}ai+10\sum_{i-1}^{nb}bi+10^{2}\sum_{i-1}^{nc}ci}{N}$$

where *a* is first-stage changes, *b* is second-stage changes, *c* is third-stage changes, *na* is the number of first-stage changes, *nb* is the number of second-stage changes, *nc* is the number of third-stage changes, and N is the number of fishes analyzed per treatment.

### 4.11. Statistical Analysis

The data were expressed as mean ± standard deviation (SD) per group. The results were evaluated using one-way ANOVA, followed by Dunnett’s post hoc test in case of significant differences (*p* < 0.05), all using GraphPad Prism (v. 5.03).

### 4.12. In Silico Analysis

SEA prediction: Inositol was evaluated through the Similarity Ensemble Approach (SEA) web server (http://sea.bkslab.org/ accessed on 23 March 2021) to investigate possible targets from carbohydrate metabolism [87]. This open server analyzes the ligands with groups of molecules that act on known receptors in its databank. Inositol’s SMILE code was inserted on the server, which gave several proteins, but only those involved in carbohydrate metabolism were selected.

Molecular docking: Based on SEA predictions and the atomic structures available in the literature, we performed a molecular docking of inositol with the enzymes maltase-glucoamylase (PDB ID: 2QMJ, 1.9 Å) and β-galactosidase (PDB ID: 3THC, 1.8 Å), using the software GOLD (v. 2020.1). The program calculates simulations between flexible targets and ligands using a genetic algorithm [108].

The coordinates used for the fitting were x = −21.78, y = −6.80, and z = −7.25 for maltase-glucoamylase and x = −3.41, y = −6.97, and z = 7.14 for β-galactosidase, using a radius of 10 Å. The crystallographic structures were previously processed by removing the cocrystallized ligand, ions, and water molecules; then, hydrogen atoms were added.

To simulate the interactions between ligand and receptors, we used the enzymes’ active site. For maltase-glucoamylase, the active site was the amino acids Asp327, Asp542, His600, Arg526, Asp443, Tyr299, Ile328, Ile364, Trp441, and Met444; for β-galactosidase, the active site was the amino acids Tyr83, Ala128, Glu129, Ile126, Cys127, Asn187, Tyr306, Tyr331, Tyr333, Trp273, Leu274, Tyr270, Glu188, and Glu268.

Before performing the docking, the structures from Protein Data Bank (PDB) were validated through the root mean square deviation (RMSD) using GOLD; this process indicates the accuracy of the fitting pose in relation to the crystallized proteins [109]. RMSD values are considered satisfactory when equal to or lower than 2 Å; in this study, the RMSD was 2 Å for maltase-glucoamylase and 0.5 Å for β-galactosidase.

## 5. Conclusions

The phytochemical study with the aqueous extract from the latex of *Hancornia speciosa* Gomes evidenced the occurrence of phenolic compounds, which play a role as preventive or curative agents against several diseases. In our study, the latex extract could significantly reduce the glycemic levels of diabetic zebrafish, decrease AST and ALT levels, and inhibit tissue injury in the pancreas. The treatment did not cause any significant disturbance in urea and creatinine levels. The toxicity studies showed that the extract was secure up to high doses in embryos and adult zebrafish. The hypoglycemic effect may be due to the presence of 1-O-methyl-myoinositol (bornesitol) since it is an inositol derivative. These molecules can probably interact with enzymes involved in carbohydrate metabolism, as evidenced by the in silico results.

## Figures and Tables

**Table 1:** NMR ^1^H characterization of *Hancornia speciosa* Gomes (LxHs).

Chemical Shift Displacement (ppm) *	Assignments	Area (%) (LxHs)
0.5–1.5	C–CH*_n_*	1.11
1.5–3.0	CH*_n_*–C=C; CH*_n_*–COR; CH*_n_*–COOR; CH*_n_*–N; CH*_n_*–Ph	8.80
3.0–4.5	CH*_n_*–OH; PhO–CH*_n_*; RCOO–CH*_n_*; CH_2_–NHCOR	78.39
4.5–6.0	CH*_n_*=CH	4.31
6.0–9.5	Ph–H; Ph–CH=CH–R	7.39

*n* = number of hydrogen atoms, where 1 ≤ *n* ≤ 3. * Aboulkas et al. (2017); Singh and Dhepe (2018).

**Table 2:** *Hancornia speciosa* Gomes (LxHs) NMR ^1^H and ^13^C data (400 × 100 MHz, CD_3_OD) compared to the literature.

Metabolite (Reference)	Position	LxHs		Literature		Structure
Cornoside (18)		δ_H_	δ_C_	δ_H_	δ_C_	
	1	-	69.2	-	69.2	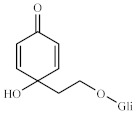
	2	7.02(^1^H, d, 10.1Hz)	154.5	7.01 (^1^H, d, 9.6Hz)	154.4	
	3	6.12 (^1^H, d, 10.2Hz)	127.8	6.11 (^1^H, d, 9.6Hz)	127.8	
	4	-	-	-	187.8	
	5	6.12 (^1^H, d, 10.2Hz)	127.9	6.11 (^1^H, d, 9.6Hz)	127.8	
	6	7.02(^1^H, d, 10.1Hz)	154.5	7.01 (^1^H, d, 9.6 Hz)	154.3	
	7	-	-	2.04 (2H, t, 6.4 Hz)	41	
	8	-	65.7	3.99 (^1^H, dt, 10.0 e 6.4 Hz) e 3.63 (^1^H, dt, 10.0 e 6.4Hz)	65.7	
	1′	-	104.2	4.21 (^1^H, d, 7.6 Hz)	104.2	
	2′	-		-	75	
	3′	-		-	77.9	
	4′	-		-	71.6	
	5′	-		-	78	
	6′	-		-	62.7	
Dihydrocornoside (19, 20)	1	-	68.5	-	68.9	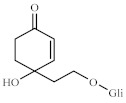
	2	6.97(^1^H, d, 10.2Hz)	157.1	6.96 (^1^H, dd, 10,0 e 1.0 Hz)	155.9	
	3	5.86 (^1^H, d, 10.1Hz)	128.4	5.86 (^1^H, d, 10.0 Hz)	127.6	
	4	-	202.2	-	198.8	
	5	-	35.3	2.47 (^1^H, ddd, 17.0, 11.5 e 5.0) e 2.55 (^1^H, ddd, 17.0, 6.5 e 5.0)	35.1	
	6	-	36	2.19-2.26 (^1^H, m) e 1.95-2.05 (^1^H, m)	36.2	
	7	-	39.9	2025-2103 (2H, m)	40	
	8	-	66.3	4.14 (^1^H, dt, 10.0 e 6.0) e 3.77 (^1^H, dt, 10.0 e 6.0)	65.9	
	1′	-	104.2	4.28 (^1^H, d, 7.5)	104.6	
	2′	-	-	3.15 (^1^H, dd, 9.0 e 7.5)	75	
	3′	-	-	-	78.5	
	4′	-	-	-	71.6	
	5′	-	-	3.64 (3H, m, H-5′ e H-6′a)	78.4	
	6′	-	-	3.86 (2H, ddl, 12.0 e ca. 1.0) e 3.64 (3H, m)	62.6	
1-O-Methyl-myoinositol (23)	1	3.01 (dd, 9.7 e 2.8 Hz)	82.9	3.08 (dd, 10.0 e 3.0 Hz)	83.2	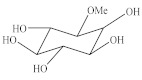
	2	4.18 (t, 2.7 Hz)	69.5	4.18 (t, 3.0 Hz)	69.8	
	3	3.33 (dd, 9.8 e 2.7 Hz)	73.1	3.37 (dd, 10.0 e 3.0 Hz)	73.4	
	4	3.61(t, 9.5 Hz)	74	3.52 (t, 10.0 Hz)	74.2	
	5	3.18 (t, 9.2 Hz)	76.2	3.15 (t, 10.0 Hz)	76.6	
	6	3.66 (t, 9.6 Hz)	74	3.49 (t, 10.0 Hz)	73.6	
	O-Me	3.44 s	57.6	3.31 s	57.8	

**Table 3:** Overview of teratogenic and lethal effects of LxHs on zebrafish embryos at 96 hpf.

Feature		CS	CD	C1	C2	C3	C4	C5	Ʃ_t_	%
Teratogenesis	Cardiac edema	0	0	0	0	0	0	0	0	0.0
	Tail malformation	0	0	0	0	0	1	2	3	60
	Scoliosis	0	0	0	0	0	1	1	2	40
	Yolk edema	0	0	0	0	0	0	0	0	0
	Growth retardation	0	0	0	0	0	0	0	0	0
Lethal embryos		0	0	4	4	5	8	12	n/a	n/a
Ʃ Teratogenic embryos		0	0	0	0	0	2	3	5	n/a
% Teratogenic embryos		0	0	0	0	0	3.3	5	n/a	n/a
% Lethal embryos		0	0	6.6	6.6	8.3	13.3	20	n/a	n/a

n/a: not applicable.

**Table 4:** SEA outputs of inositol compared to enzymes involved in carbohydrate metabolism.

Enzyme	E-Value	MaxTc
β-galactosidase	9.90^−17^	0.29
Intestinal maltase-glucoamylase	1.91^−10^	0.33
α-galactosidase	3.56^−32^	0.33
Bovine α-L-fucosidase	2.23^−20^	0.33
Rodent α-L-fucosidase	7.14^−20^	0.33
Glucosylceramidase	1.11^−16^	0.45
Lysosomal acid α-glucosidase	2.39^−11^	0.33
Glycogen debranching enzyme	4.47^−10^	0.33
Intestinal sucrase-isomaltase	1.02^−6^	0.33
α-galactosidase	3.30^−6^	0.33

**Table 5:** Teratogenic and lethal effects observed in zebrafish embryos across the developmental time.

Developmental Toxicity		24 hpf	48 hpf	72 hpf	96 hpf
Lethal effects	Coagulated eggs ^a^	+	+	+	+
	Lack of somite formation	+	+	+	+
	Lack of tail displacement	+	+	+	+
	No heartbeat ^b^	+	+	+	+
Teratogenic effects	Yolk edema	+	+	+	+
	Growth retardation	+	+	+	+
	Tail malformation		+	+	+
	Cardiac edema		+	+	+
	Scoliosis			+	+

^a^ Coagulated eggs are milky white and appear dark on the optical microscope. ^b^ Lack of heartbeat for at least one minute.

**Table 6:** Tissue changes used to calculate the index of histological changes in zebrafish pancreas.

Tissue Changes	Stage
Loss of cellular structure	I
Pyknotic nuclei	I
Nuclei fragmentation	I
Presence of natural killer cells	I
Presence of macrophages	I
Presence of lymphocytes	I
Insulitis	II
Cytoplasm degeneration	II
Nuclei decomposition	II
Islets atrophy	II
Islets absence	II
Acinar cell atrophy	II
Necrosis	III

## Data Availability

The data presented in this study are available in the article.

## References

[1] Filho V.C., Zanchett C.C.C. (2020). Fitoterapia Avançada: Uma Abordagem Química, Biológica e Nutricional.

[2] Pedrosa V.M.D. (2018). Fisiologia, Qualidade e Potencial Funcional de Frutos de Diferentes Acessos de Mangabeira (Hancornia speciosa Gomes).

[3] Brasil, Ministério do Meio Ambiente–MMA Relatório Técnico de Monitoramento do Desmatamento no Bioma Cerrado, 2001 a 2008: Dados Revisados. CENTRO DE SENSORIAMENTO REMOTO-CSR/IBAMA-Nov. 2009 DF. http://www.mma.gov.br/estruturas/sbf_chm_rbbio/_arquivos/relatorio_tecnico_monitoramento_desmate_bioma_cerrado_csr_rev_72_72.pdf.

[4] Honda N.K., Garcez W.S., Garcez F.R., Conceição C.A. (1990). Estudo químico de plantas de Mato Grosso do Sul I: Triagem fitoquímica. Rev. Científica Cult. UFMS.

[5] Brandão G.C., Kroon E.G., Dos Santos J.R., Stehmann J.R., Lombardi J.A., de Oliveira A.B. (2011). Antiviral activity of plants occurring in the state of minas gerais (Brazil): Part III. J. Chem. Pharm. Res..

[6] Assumpção C.F., Bachiega P., Morzelle M.C., Nelson D.L., Ndiaye E.A., Rios A.O.S., Souza É.C. (2014). Caracterização, potencial antioxidante e estudo citotóxico de frutas mangaba. Ciência Rural.

[7] Lima N.G.A., Kaffashi S., Luiz W.T., Ferreira W.R., Dias S.Y.S.A., Pazin G.V., Violante I.M.P. (2015). Quantificação de metabólitos secundários e avaliação da atividade antimicrobiana e antioxidante de algumas plantas selecionadas do Cerrado de Mato Grosso. Rev. Bras. Plantas Med..

[8] Moraes T.M., Rodrigues C.M., Kushima H., Bauab T.M., Villegas W., Pellizzon C.H., Brito A.R., Hiruma-Lima C.A. (2008). Hancornia speciosa: Indications of gastroprotective, healing and anti-Helicobacter pylori actions. J. Ethnopharmacol..

[9] Neves J.S., Franchin M., Rosalen P.L., Omar N.F., Santos M.A., Paschoal J.A., Novaes P.D. (2016). Evaluation of the osteogenic potential of Hancornia speciosa latex in rat calvaria and its phytochemical profile. J. Ethnopharmacol. Limerick.

[10] Almeida L.M., Floriano J.F., Ribeiro T.P., Magno L.N., Da Mota L.S.L.S., Peixoto N., Mrué F., Melo-Reis P.R., Lino R.S., Graeff C.F.O. (2014). Hancornia speciosa latex for biomedical applications: Physical and chemical properties, biocompatibility assessment and angiogenic activity. J. Mater. Sci. Mater. Med..

[11] Floriano J.F., Neto F.C., Da Mota L.S.L.S., Ferreira R.S., Gonçalves P., Borges F.A., Graefs C.F.O., Neto F.C., Furtado E.L., Barraviera B. (2016). Comparative study of accelerated regeneration of bone tissue by latex membranes of Hevea brasiliensis and Hancornia speciosa. Biomed. Phys. Eng. Express.

[12] Ribeiro T.P., Sousa T.R., Arruda A.S., Peixoto N., Gonçalves P.J., Almeida L.M. (2016). Evaluation of cytotoxicity and genotoxicity of Hancornia speciosa latex in Allium cepa root model. Braz. J. Biol..

[13] Santos A.D.C. (2012). Desenvolvimento e Validação de Métodos por HPLC-DADELSD Para Controle de Qualidade Químico do látex do Caule e do Fruto de Mangaba (Hancornia speciosa GOMES), Dissertação de Mestrado.156 f. Sergipe, Brazil. https://www.researchgate.net/publication/299484431.

[14] Marinho D.G., Alviano D.S., Matheus M.E., Alviano C.S., Fernandes P.D. (2011). The latex obtained from Hancornia speciosa Gomes possesses anti-inflammatory activity. J. Ethnopharmacol..

[15] Santos I.V.F., Souza G.C., Santana G.R., Duarte J.L., Fernandes C.P., Keita H., Velázquez-Moyado J.A., Navarrete A., Ferreira I.M., Carvalho H.O. (2018). Histopathology in Zebrafish (Danio rerio) to Evaluate the Toxicity of Medicine: An Anti-Inflammatory Phytomedicine with Janaguba Milk (Himatanthus drasticus Plumel).

[16] Souza G.C., Pereira A.C.M., Viana M.D., Ferreira A.M., Silva I.D.R., Oliveira M.M.R., Barbosa W.L.R., Silva L.B., Ferreira I.M., Santos C.B.R. (2019). *Acmella oleracea* (L) R. K. Jansen Reproductive Toxicity in Zebrafish: An In Vivo and In Silico Assessment. Evid.-Based Complementary Altern. Med..

[17] Hyacienth B.M.S., Picanço K.R.T., Sánchez-Ortiz B.L., Silva L.B., Pereira A.S.M., Góes L.D.M., Borges R.S., Ataíde R.C., Santos C.B.R., Carvalho H.O. (2020). Hydroethanolic extract from Endopleura uchi (Huber) Cuatrecasas and its marker bergenin: Toxicological and pharmacokinetic studies in silico and in vivo on zebrafish. Toxicol. Rep..

[18] Sociedade Brasileira de Diabetes (2019). Posicionamento Oficial SBD nº 01/2019–Conduta Terapêutica No Diabetes Tipo 2: Algoritmo SBD 2019.

[19] Centers for Disease Control Prevention Diabetes 2014. Report Card. Cdc, v. TTY. 232-4636. www.cdc.333/diabetes/library/reports/congress.html.

[20] Miranda J.C.M.M. (2018). Investigação Molecular do Mecanismo de Ação Antidiabética da Nanodispersão de uma Fração Flavonoídica de Baccharis Reticularia. Dissertação (Mestrado em Ciências da Saúde)–Programa de pós-graduação em Ciências Farmacêuticas. Master’s Thesis.

[21] Rocha F.A.G., Araújo M.F.F., Costa N.D.L., Silva R.P. (2015). O uso terapeutico da flora na história mundial. Holos.

[22] Santos A.C.B., Silva M.A.P., Santos M.A.F., Leite T.R. (2013). Levantamento etnobotânico, químico e farmacológico de espécies de Apocynaceae Juss. ocorrentes no Brasil. Rev. Bras. Plantas Med..

[23] Cercato L.M., White P.A.S., Nampo F.K., Santos M.R., Camargo E.A. (2015). A systematic review of medicinal plants used for weight loss in Brazil: Is there potential for obesity treatment?. J. Ethnopharmacol..

[24] Pereira A.C., Pereira A.B.D., Moreira C.C., Botion L.M., Lemos V.S., Braga F.C., Cortes S.F. (2015). Hancornia speciosa Gomes (Apocynaceae) as a potential anti-diabetic drug. J. Ethnopharmacol..

[25] Neto L.S., Moraes-Souza R.Q., Soares T.S., Pinheiro M.S., Leal-Silva T., Hoffmann J.C., Américo M.F., Campos K.E., Damasceno D.C., Volpato G.T. (2020). A treatment with a boiled aqueous extract of Hancornia speciosa Gomes leaves improves the metabolic status of streptozotocin-induced diabetic rats. BMC Complement. Med. Ther..

[26] Hung H.Y., Qian K., Morris-Natschke S.L., Hsu C.S., Lee K.H. (2012). Recent discovery of plant-derived anti-diabetic natural products. Nat. Prod. Rep..

[27] Bizzarri M., Carlomagno G. (1978). Inositol: History of an effective therapy for polycystic ovary syndrome. Eur. Rev. Med. Pharmacol. Sci..

[28] Berggren P.O., Barker C.J. (2008). A key role for phosphorylated inositol compounds in pancreatic beta-cell stimulus-secretion coupling. Adv. Enzyme Regul..

[29] Endringer D.C., Pezzuto J.M., Soares C.M., Braga F.C. (2007). L-(+)-Bornesitol. Acta Crystallogr. Seção E Relatórios Estrut. Online.

[30] Da Silva E.C. (2018). Estudo de Padronização de Extratos de Hancornia speciosa Gomes Como Alternativa Terapêutica Para Obesidade. 100f. Dissertação (Mestrado)-Universidade Federal do Maranhão, São Luís. https://tedebc.ufma.br/jspui/handle/tede/tede/2193.

[31] Souza G.C., Duarte J.L., Fernandes C.P., Velázquez-Moyado J.A., Navarrete A., Carvalho J.C.T. (2016). Obtainment and study of the toxicity of perillyl alcohol nanoemulsion on zebrafish (Danio rerio). J. Nanomed. Res..

[32] Carvalho J.C.T., Keita H., Santana G.R., Souza G.C., Santos I.V.F., Amado J.R.R., Kourouma A., Prada A.L., Carvalho H.O., Silva M.L. (2017). Effects of Bothrops alternatus venom in zebrafish: A histopathological study. Inflammopharmacology.

[33] Borges S.B., Keita H., Sanchez-Ortiz B.L., Sampaio T.I.S., Ferreira I.M., Lima E.S., Silva M.J.A., Fernandes C.P., Oliveira A.E.M.F.M., Conceição E.C. (2018). Anti-inflammatory activity of nanoemulsions of essential oil from Rosmarinus officinalis L.: In vitro and in zebrafish studies. Inflammopharmacology.

[34] Melo N.C., Sánchez-Ortiz B.L., Sampaio T.I.S., Pereira A.C.M., Silva Neto F.L.P., Silva H.R., Cruz R.A.S., Keita H., Pereira A.M.S., Carvalho J.C.T. (2019). Anxiolytic and Antidepressant Effects of the Hydroethanolic Extract from the Leaves of Aloysia polystachya (Griseb.) Moldenke: A Study on Zebrafish (Danio rerio). Pharmaceuticals.

[35] Custódio de Souza G., Dias Ribeiro da Silva I., Duarte Viana M., Costa de Melo N., Sánchez-Ortiz B.L., Maia Rebelo de Oliveira M., Ramos Barbosa W.L., Maciel Ferreira I., Tavares Carvalho J.C. (2019). Toxicidade Aguda do Extrato Hidroetanólico das Flores de Acmella oleracea L. em Peixe-zebra (Danio rerio): Estudos Comportamentais e Histopatológicos. Pharmaceuticals.

[36] Pereira A.C.M., Sánchez-Ortíz B.L., de Melo E.L., Hage-Melim L.I.D.S., Borges R.S., Hu X., Carvalho J.C.T. (2021). Perillyl alcohol decreases the frequency and severity of convulsive-like behavior in the adult zebrafish model of acute seizures. Naunyn-Schmiedeberg’s Arch. Pharmacol..

[37] Biemar F., Argenton F., Schmidtke R., Epperlein S., Peers B., Driever W. (2001). Pancreas development in zebrafish: Early dispersed appearance of endocrine hormone expressing cells and their convergence to form the definitive islet. Dev. Biol..

[38] Royer M., Herbette G., Eparvier V., Beauchêne J., Thibaut B., Stien D. (2010). Secondary metabolites of Bagassa guianensis Aubl. wood: A study of the chemotaxonomy of the Moraceae Family. Phytochemistry.

[39] Lopes W.A., Fascio M. (2004). Esquema para interpretação de espectros de substâncias orgânicas na região do infravermelho. Quim. Nova.

[40] Silverstein R.M., Webster F.X., Kiemle D.J. (2006). Identificação Espectrométrica de Compostos Orgânicos.

[41] Pavia D.L., Lampiman G.M., Kriz G.S., Vyvyan J.R. (2010). Introdução à Espectroscopia.

[42] Rupasinghe H.P.V.Z. (2014). Application of NMR Spectroscopy in Plant Polyphenols Associated with Human Health. Application of NMR Spectroscopy in Food Science.

[43] Jia J., Zhang F., Li Z., Qin X., Zhang L. (2015). Comparison of Fruits of Forsythia suspensa at Two Different Maturation Stages by NMR-Based Metabolomics. Molecules.

[44] Singh S.K., Dhepe P.L. (2018). Experimental evidences for existence of varying moieties and functional groups in assorted crop waste derived organosolv lignins. Ind. Crop. Prod..

[45] Jensen S.R. (2000). Chemical relationships of Polypremum procumbens, Tetrachondra hamiltonii and Peltanthera floribunda. Biochem. Syst. Ecol..

[46] Verdan M.H., Souza L.M., Carvalho J.E., Costa D.B.V., Salvador M.J., Barison A., Stefanello M.E.A. (2015). Two new hydronaphthoquinones from Sinningia aggregata (Gesneriaceae) and cytotoxic activity of aggregatin D. Chem. Biodivers..

[47] Kim D.H., Han K.M., Bang M.H., Lee Y.H., Chung I.S., Keun H.D., Kim D.K., Kim S.H., Kwon B.M., Park M.H. (2007). Cyclohexylethanoids from the Flower of Campsis grandiflora. Bull. Korean Chem. Soc..

[48] Winiewski W. (2016). Constituintes Químicos e Atividade Antimicrobiana de Sinnningia Warmingii (GESNERIACEAE). Master’s Thesis.

[49] Kuwajima H., Takai Y., Takaishi K., Inoue K. (1998). Synthesis of 13C-Labeled Possible Intermediates in the Biosynthesis of Phenylethanoid Derivatives, Cornoside and Rengyosides. Chem. Pharm. Bull.

[50] Hase T., Kawamoto Y., Ohtani K., Kasai R., Yamasaki K., Picheansoonthon C. (1995). Cyclohexylethanoids and related glucosides from millingtonia hortensis. Phytochemistry.

[51] Shevts V.I. (1974). The chemistry of myoinositol. Russ. Chem. Rev..

[52] Angyal S.J., Odier L. (1983). The effect of O-methylation on chemical shifts in the 1H- and 13CN.M.R. spectra of cyclic. Carbohydrate Res..

[53] Sureshan K.M., Shasidar M.S., Praveen T., Das T. (2003). Regioselective protection and deprotection of inositol hydroxyl groups. Chem. Prev..

[54] Almeida M.V., Couri M.R.C., Assis J.V., Anconi C.P.A., Santos H.F., Almeida W.B. (2012). 1H NMR analysis of O-methyl-inositol isomers: A joint experimental and theoretical study. Magn. Reson. Chem..

[55] Santos H.F., Chagas M.A., Souza L.A., Rocha W.R., Almeida M.V., Anconi C.P.A., Almeida W.B. (2017). Water Solvent Effect on Theoretical Evaluation of 1 H NMR Chemical Shifts: O Methyl-Inositol Isomer. J. Phys. Chem..

[56] Zhang X., Li C., Gong Z. (2014). Development of a Convenient In Vivo Hepatotoxin Assay Using a Transgenic Zebrafish Line with Liver-Specific DsRed Expression. PLoS ONE.

[57] Zhang Y., Liu K., Hassan H.M., Guo H., Ding P., Han L., He Q., Chen W., Hsiao C.-D., Zhang L. (2016). Liver Fatty Acid Binding Protein Deficiency Provokes Oxidative Stress, Inflammation, and Apoptosis-Mediated Hepatotoxicity Induced by Pyrazinamide in Zebrafish Larvae. Antimicrob. Agents Chemother..

[58] Giannini E.G., Testa R., Savarino V. (2005). Alteração das enzimas hepáticas: Um guia para medicos. CMAJ.

[59] Zimmermann S., Gruber L., Schlummer M., Smolic S., Fromme H. (2012). Determination of phthalic acid diesters in human milk at low ppb levels. Food Addit. Contam. Part. A Chem. Anal. Control. Expo. Risk Assess..

[60] Kitamura K., Iwasaki H.O., Yasoshima A., Yoshikawa H., Yoshikawa T., Okaniwa A. (1992). Pathology of Chemically Induced Chronic Active Hepatitis in Mice. Exp. Mol. Pathol..

[61] Huang X., Choi Y., Im H., Yarimaga O., Yoon E., Kim H. (2006). Aspartate Aminotransferase (AST/GOT) and Alamine Aminotransferase (ALT/GPT) Detection Techiniques. Sensors.

[62] Chandak N., Kumar P., Kaushik P., Varshney P., Sharma C., Kaushik D., Jain S., Aneja K.R., Sharma P. (2014). Dual evaluation of some novel 2-amino-substituted coumarinylthiazoles as anti-inflammatory–antimicrobial agents and their docking studies with COX-1/COX-2 active sites. J. Enzym. Inhib. Med. Chem..

[63] Sim L., Quezada-Calvillo R., Sterchi E.E., Nichols B.L., Rose D.R. (2008). Human Intestinal Maltase–Glucoamylase: Crystal Structure of the N-Terminal Catalytic Subunit and Basis of Inhibition and Substrate Specificity. J. Mol. Biol..

[64] Ren L., Cao X., Geng P., Bai F., Bai G. (2011). Study of the inhibition of two human maltase-glucoamylases catalytic domains by different α-glucosidase inhibitors. Carbohydr. Res..

[65] Li P.H., Lin Y.W., Lu W.C., Hu J.M., Huang D.W. (2016). In vitro hypoglycemic activity of the phenolic compounds in longan fruit (Dimocarpus Longan var. Fen ke) shell against α-glucosidase and β-galactosidase. Int. J. Food Prop..

[66] Ohto U., Usui K., Ochi T., Yuki K., Satow Y., Shimizu T. (2012). Crystal Structure of Human β-Galactosidase structural basis of GM1 gangliosidosis and morquio b diseases. J. Biol. Chem..

[67] Pani A., Giossi R., Menichelli D., Fittipaldo V., Agnelli F., Inglese E., Romandini A., Roncato R., Pintaudi B., Del Sole F. (2020). Inositol and Non-Alcoholic Fatty Liver Disease: A Systematic Review on Deficiencies and Supplementation. Nutrients.

[68] Ferreira D.Q., Oliveira A.E.M.F.M. (2019). Estudo da Atividade Antidiabética e Toxicidade Aguda do Extrato Hidroetanólico dos Frutos de Libidibia ferrea em Zebrafish (Danio rerio). Programa de Pós-Graduação em Ciências Farmacêuticas. Master’s Thesis.

[69] Castañeda R., Rodriguez I., Nam Y.H., Hong B.N., Kang T.H. (2017). Trigonelline promotes auditory function through nerve growth factor signaling on diabetic animal models. Phytomedicine.

[70] Seth A., Stemple D.L., Barroso I. (2013). O uso emergente de peixe-zebra para modelar doenças metabólicas. Dis. Model. Mech..

[71] Eames S.C., Philipson L.H., Prince V.E., Kinkel M.D. (2010). A medição do açúcar no sangue em peixes-zebra revela a dinâmica da homeostase da glicose. Zebrafish.

[72] Jurczyk A., Roy N., Bajwa R., Gut P., Lipson K., Yang C., Covassin L., Racki W.J., Rossini A.A., Phillips N. (2011). Dynamic glucoregulation and mammalian-like responses to metabolic and developmental disruption in zebrafish. Gen. Comp. Endocrinol..

[73] Maddison L.A., Chen W. (2017). Modeling pancreatic endocrine cell adapter and diabetes in the zebrafish. Frente. Endocrinol..

[74] Olsen A.S., Sarras M.P., Intine R.V. (2010). Limb regeneration is impaired in an adult zebrafish model of diabetes mellitus. Wound Repair Regen.

[75] Landgraf K., Schuster S., Meusel A., Garten A., Riemer T., Schleinitz D., Kiess W., Körner A. (2017). Short-term overfeeding of zebrafish with normal or high-fat diet as a model for the development of metabolically healthy versus unhealthy obesity. BMC Physiol..

[76] Zang L., Shimada Y., Nishimura N. (2017). Development of a Novel Zebrafish Model for Type 2 Diabetes Mellitus. Sci. Rep..

[77] Rodrigues G.M., Nascimento F.G.O., Bizare A., Oliveira W.J., Guimarães E.C., Mundim A.V. (2018). Serum Biochemical Profile of Nile Tilapias (*Oreochromis niloticus*) Bred in Net Cages during Summer and Winter. Acta Sci. Vet..

[78] Thrall M.A., Baker D.C., Campbell T.W., DeNicola D., Fettman M.J., Lassen E.D., Rebar A., Weiser G. (2006). Hematologia e Bioquímica Clínica Veterinária.

[79] Helfman G.S., Collette B.B., Facey D.E. (1997). The Diversity of Fishes.

[80] Rotta M.A. (2003). Aspectos Gerais da Fisiologia e Estrutura do Sistema Digestivo dos Peixes Relacionados à Pisciculture.

[81] Ward A.B., Warga R.M., Prince V.E. (2009). Origino f the zebrafish endocrine and exocrine pancreas. Proc. Natl. Acad. Sci. USA.

[82] Holden J.A., Layfield L.L., Matthews J.L. (2012). The Zebrafish: Atlas of Macroscopic and Microscopic Anatomy.

[83] Benchoula K., Khatib A., Quzwain F., Che Mohamad C.A., Wan Sulaiman W., Abdul Wahab R., Ahmed Q.U., Abdul Ghaffar M., Saiman M.Z., Alajmi M.F. (2019). Otimização da indução hiperglicêmica em peixes-zebra e avaliação do nível de glicose no sangue e da impressão digital de metabólitos tratados com extrato de folha de Jack de Psychotria malayana. Molecules.

[84] Cotran R.S., Kumar V., Robbins S.L. (2000). Patologia Estrutural e Funcional.

[85] Roberts R.J. (1989). Fish Pathology.

[86] Keiser M., Roth B.L., Armbruster B.N., Ernsberger P., Irwin J., Shoichet B.K. (2007). Relating protein pharmacology by ligand chemistry. Nat. Biotechnol..

[87] Glickman N.S., Yelon D. (2002). Cardiac development in zebrafish: Coordination of form and function. Semin. Cell Dev. Biol..

[88] Mu X., Chai T., Wang K., Zhu L., Huang Y., Shen G., Li Y., Li X., Wang C. (2016). The developmental effect of difenoconazole on zebrafish embryos: A mechanism research. Environ. Pollut..

[89] Wang S., Liu K., Wang X., He Q., Chen X. (2011). Toxic effects of celastrol on embryonic development of zebrafish (Danio rerio). Drug Chem. Toxicol..

[90] He Q., Liu K., Wang S., Hou H., Yuan Y., Wang X. (2012). Toxicity induced by emodin on zebrafish embryos. Drug Chem. Toxicol..

[91] Ribeiro L.C. (2013). Investigação do Efeito Ictiotóxico do Extrato Etanolico da Raiz de *Spilanthes acmella* (jambu) em Zebrafish Através da Análise Eletrofisiológica e Comportamental. Master’s Thesis.

[92] Goksøyr A. (1995). Use of cytochrome P450 lA (CYP1A) in fish as a biomarker of aquatic pollution. Arch. Toxicol. Suppl..

[93] Borges R.B., Souza G.C., Ferreira A.C.M., Carvalho J.C.T. (2019). Use of Zebrafish (Danio rerio) in Non-Clinical Toxicological Studies of New Drugs.

[94] Vliegenthart A.D., Tucker C.S., Del Pozo J., Dear J.W. (2014). Zebrafish as model organisms for studying drug-induced liver injury. Br. J. Clin. Pharmacol..

[95] Alvarez-Pellitero P., Sitja-Bobadilla A. (2011). Pathology of Myxosporea in marine fish culture. Dis. Aquat. Org..

[96] Roberts R.J., Ellis A.E., Roberts R.J. (2012). The anatomy and physiology of teleosts. Fish Pathology.

[97] Takashima F., Hibiya T. (1984). An Atlas of Fish Histology-Normal and Pathological Features.

[98] Arruda A.S., Faria R.Q., Peixoto N., Moreira A.S.F.P., Floriano J.F., Graeff C.F.O., Gonçalves P.J., Almeida L.M. (2016). Avaliação da produção de látex em mangabeiras do cerrado goiano. Ciência Florestal..

[99] Aboulkas A., Hammani H., El Achaby M., Bilal E., Barakat A., El Harfi K. (2017). Valorization of algal waste via pyrolysis in a fixed-bed reactor: Production and characterization of bio-oil and bio-char. Bioresour. Technol..

[100] Ferreira M.F.P., Oliveira B.F.H., Pinheiro W.B.S., Correa N.F., França L.F., Ribeiro N.F.P. (2020). Generation of biofuels by slow pyrolysis of palm empty fruit bunches: Optimization of process variables and characterization of physical-chemical products. Biomass Bioenergy.

[101] Ge Y., Sun M., Salomé-Abarca L.F., Wang M., Choi Y.H. (2018). Investigation of species and environmental effects on rhubarb roots metabolome using 1H NMR combined with high performance thin layer chromatography. Metabolomics.

[102] Salomé-Abarca L.F., van der Pas J., Kim H.K., van Uffelen G.A., Klinkhamer P.G., Choi Y.H. (2018). Metabolic discrimination of pine resins using multiple analytical platforms. Phytochemistry.

[103] Leary S., Anthony R., Cartner S., Corey D., Grandin T., Greenacre C., Gwaltney-Brant S., McCrackin M.A., Meyer R., Miller D. (2013). AVMA Guidelines for the Euthanasia of Animals.

[104] Cueva-Quiroz V.A., Yunis-Aguinaga J., Ramos-Espinoza F., Claudiano G.S., Marinho-Neto Fausto A., Abreu S.A., Moraes F.R., Moraes J.R.E. (2014). Protocolo Para Indução de Diabetes Com Aloxana em Pacu Piaractus Mesopotamicus. Centro de Aquicultura da Unesp, CAUNESP, Jaboticabal, SP. https://www.pesca.sp.gov.br/12recip/Resumos_PDFs/PROTOCOLO_INDUCAO_DIABETES_ALOXANA_PACU_Piaractus_mesopotamicus.pdf.

[105] Favoretto S.M., Seabra D.I., Olivato M.C.M. (2019). Guia de Eutanásia Para Animais de Ensino e Pesquisa.

[106] Dorsemans A.C., D’Hellencourt C.L., Ait-Arsa I., Jestin E., Meilgac O., Diotel N. (2017). Acute and Chronic Models of Hyperglycemia in Zebrafish: A Method to Assess the Impact of Hyperglycemia on Neurogenesis and the Biodistribution of Radiolabeled Molecules. J. Vis. Exp..

[107] Poleksic V., Mitrovic-Tutundzic V., Müller R., Lloyd R. (1994). Fish gills as a monitor of sublethal and chronic effects of pollution. Sublethal and Chronic Efects of Pollutants on Freshwater Fish.

[108] Pagadala N.S., Syed K., Tuszynski J. (2017). Software for molecular docking: A review. Biophys. Rev..

[109] Cole J.C., Nissink J.W.M., Taylor R. (2005). Protein–ligand docking and virtual screening with GOLD. Virtual Screening in Drug Discovery.

